# Are you really smiling? Display rules for emojis and the relationship between emotion management and psychological well-being

**DOI:** 10.3389/fpsyg.2023.1035742

**Published:** 2023-03-03

**Authors:** Moyu Liu

**Affiliations:** Graduate School of Interdisciplinary Information Studies, The University of Tokyo, Tokyo, Japan

**Keywords:** emojis, display rules, emotion norms, emotional expression, social context, well-being

## Abstract

Display rules specify socially appropriate facial expressions in a given situation. However, managing emotions for such a social adaption sometimes leads to deleterious psychological outcomes. Given that people nowadays rely on emojis to express emotions online, the present study investigated (1) whether display rules exist in emoji communications and (2) how emotion management using emojis is associated with psychological well-being. Prior studies have demonstrated the effects of context on the frequency of emoji use. However, the intensity and type of expression may differ, even if emojis are used at the same frequency. The current study thus investigated whether emotional expressions and the types of emojis used are adjusted to contexts similar to facial displays. As many as 1,289 Japanese participants typed emojis in response to Internet chats and reported the intensity of their emotional expressions. The contexts of the chats varied depending on the target of use, the emotional value of contexts, and private or public settings. The results showed that, similar to facial displays, individuals expressed emotions through emojis more with those closely related, more in positive contexts than in negative contexts, and more in private than in public contexts. When the expressions were intense, individuals used emojis consistent with the emotional value of the context. Upon attenuating the expressions, this study found that individuals tended to use euphemistic emojis and sent smiling emojis in negative contexts to manage the expressions. Moreover, expressing emotions with emojis was associated with subjective well-being, whereas managing emotions with emojis was weakly associated with depressive symptoms. Together, this study indicates the existence of display rules for emojis, calling for future research on the psychological impact of online emotion norms.

## 1. Introduction

In face-to-face (FTF) communication, people adjust their emotional expressions to social contexts following facial display rules. For example, individuals will display positive expressions even if they receive an unwanted gift, as the standard display rules require individuals “to look pleased when someone gives you something” (Goffman, 1967, as cited in Saarni 1984). As such, display rules govern the appropriateness of expressive behavior, regulate the type and intensity of emotions expressed in certain situations (Fussell, 2002), and vary according to culture (Ekman and Friesen, 1969; Ekman, 1970; Ekman, 1972). Numerous studies have indicated the influence of social contexts on emotional expression (Fridlund, 1991; Friedman and Miller-Herringer, 1991; Fischer et al., 2003). Furthermore, scholars have argued that different social contexts apply context-specific display rules that dictate socially appropriate expressions (Derks et al., 2008). Under these rules, expressive displays appear to be amplified with a friend than with a stranger (Wagner and Smith, 1991; Jakobs et al., 2001), and individuals show more pleasure at winning a competition when they are alone than when they are with others (Friedman and Miller-Herringer, 1991).

With the advent and prevalence of communication technology, interaction is increasingly moving from FTF to digital contexts (Ministry of Internal Affairs and Communications of Japan, 2021). Although computer- or mobile-based communication (CMC) was initially considered an impersonal and emotionless medium because of the lack of nonverbal cues (Sproull and Kiesler, 1986; Rice and Love, 1987), subsequent studies have argued that emotions are abundant in CMC, especially since emojis serve the function of expressing emotions similar to nonverbal displays (Walther and D’Addario, 2001; Derks et al., 2008; Boutet et al., 2021). Emojis were initially invented by Shigetaka Kurita, a Japanese individual (Alt, 2016). To date, over 90% of Internet users worldwide use emojis (Emogi, 2015), which also constitute the main expression tools for daily communication among young Japanese individuals (Kato, 2017). By definition, emojis are graphic symbols used in online communication, which can represent facial expressions, emotions, animals, weather, and so on. Many studies have acknowledged that emojis can express or enhance emotions on the Internet, similar to facial displays in FTF communication (Lu et al., 2016; Bai et al., 2019; Boutet et al., 2021). Specifically, evidence suggests that emojis can accentuate emotional tone, clarify the intention, and express subtle moods (Derks et al., 2008; Lo, 2008; Luor et al., 2010). As emotional emblems on the Internet, emojis have also been applied effectively in sentiment analysis to explore the digital emotion culture (Cahyaningtyas et al., 2017; Felbo et al., 2017). Furthermore, Fischer and Herbert (2021) argued that, as opposed to emoticons, which are composed of alphanumeric and punctuation characters, emojis can convey emotional sentiments more accurately. Importantly, they revealed that emojis can represent affective content similar to facial expressions, and for some specific emotions, emojis are even more expressive. The general functional equivalence of emojis and facial expressions in expressing emotions was also verified very recently through a series of experiments (Erle et al., 2021).

Since emojis can express emotions in CMC similarly to facial expressions in FTF, the question is whether display rules also exist in emoji communication. That is, are emotional expressions using emojis adjusted to social contexts in the same way as facial expressions? Despite the rapid growth of cyberspace, the rules of emotional expression in digital communication remain poorly understood (Cheshin, 2020). A few studies have provided information on the contextual demands of emoji use (Derks et al., 2007; Kaye et al., 2016; Glikson et al., 2018). For example, using a smiley face in a formal work email is considered inappropriate (Glikson et al., 2018). However, as opposed to focusing on emotion norms, most existing studies address some basic issues regarding how emojis are used (Coyle and Carmichael, 2019; Jones et al., 2020; Liu and Sun, 2020) and their functions (Walther and D’Addario, 2001; Luor et al., 2010; Al Rashdi, 2018). Online communication has created new rules, most of which are implicit and not written anywhere (McLaughlin and Vitak, 2012). However, violating these rules often leads to detrimental outcomes such as bad first impressions (Glikson et al., 2018), stigmatization, and marginalization (Grattet, 2011). As digital media has been integrated into the lives of adolescents and impacts them greatly (Manago and McKenzie, 2022), exploring these rules is crucial and meaningful. With this in mind, the first aim of this study was to (1) investigate whether emotional expression using emojis is adjusted to the social context, similar to facial displays.

As a social etiquette, display rules can create a desirable emotional atmosphere (Gabriel et al., 2016) and maintain a social hierarchy (Dzokoto et al., 2018). Also, displaying socially appropriate emotions can have a positive impact on a displayer (Cheshin, 2020). Nevertheless, managing emotional expressions in light of social norms often means that the intensity of emotional expressions does not reflect the actual intensity of the experienced emotions. Drawing upon Goffman’s (1959) impression management theory, Hochschild (1983) originally defined emotion management as the modulation or management of a person’s emotional expressions to align with the display rules of the social context or roles and differentiated emotion management carried out in a public context (emotion labor) from that in private social life (emotion work). She also criticized that under the constraints of emotion norms, individuals will exaggerate or suppress their emotions to ensure that their expressions are considered appropriate, which can lead to harmful emotional alienation and uncomfortable inconsistencies between displayed and felt emotions. This alert has also triggered studies focusing on the association between different types of emotion management and psychological well-being (Grandey, 2003; Mesmer-Magnus et al., 2012; Lee et al., 2016). For example, a recent study has reported that dealing with display rules may increase the risk of depression (Chun et al., 2020). However, in an earlier literature review, both the positive and negative effects of emotion management were pointed out, such as enhancing personal accomplishment, and causing deleterious psychological consequences, including emotional exhaustion, depersonalization, or psychosomatic complaints (Zapf, 2002). Importantly, Riordan (2017) noted that with the prevalence of digital communication, emotion management has infiltrated our lives to a greater extent than ever before, wherein emojis can be used as digital emotion work tools, helping individuals manage the expressions to fulfill different social roles. These findings raise the pertinent question of how the use of emojis to manage emotional expression is associated with mental health. However, despite existing studies on emojis, the association between emotion management with emojis and mental health remains unclear. Thus, the second aim of the present study was to (2) analyze the relationship between emotion management with emojis and psychological well-being.

To achieve these two objectives, prior research on the display rules of facial expressions and how social contexts affect emoji use were summarized and compared to formulate specific hypotheses. Thereafter, the relationship between emotion management and mental health was outlined for further observation.

## 2. Literature review and hypothesis development

### 2.1. Facial display rules

Emotional expression, since it occurs during a certain interaction in a social situation, is regulated by display rules that specify socially appropriate expressions in a given situation (Derks et al., 2007). Such display rules were initially used to explain cultural differences in facial expressions. For example, culture X might exhibit upturned lips at a funeral, whereas culture Y depicts downturned lips (Ekman, 1970). In addition to culturing, Greenaway et al. (2018) and Cheshin (2020) pointed out other elements related to the appropriateness of emotional displays. Particularly, these can be categorized as displayers (e.g., age, gender), display targets (e.g., a friend or a high-status person), social setting (e.g., public or private), and the displayed emotion (e.g., whether it is positive or negative).

Displayers’ characteristics can lead to different expectations of emotional displays. Among them, gender comprises an important factor that influences the appropriateness of the presented emotions (Cheshin, 2020). Women are expected to be more caring and tender than men (Shields, 2005), whereas men are expected to restrain their affective behaviors relative to women (Mesch and Beker, 2010). Thus, individuals perceive affectionately emotive females as being more appropriate than affectionately emotive males (Weisberg et al., 2011). More specifically, a male’s display of anger would be considered more appropriate than a female’s, whereas a female’s display of sadness and happiness would be considered more appropriate than a male’s (Timmers et al., 1998; Sloan, 2012). Matsumoto et al. (1998) similarly reported that women exert more control on anger and disgust, whereas men exert more control on fear and surprise.

Display targets can also shape the expression of emotions. Consistent results have been shown that people are more likely to smile and show facial expressions with their friends, whereas they suppress their emotions in the presence of strangers (Wagner and Smith, 1991; Jakobs et al., 2001; Lee and Matsumoto, 2011; Pollastri et al., 2018). Additionally, this expression pattern has been confirmed across cultures, indicating an increase in emotional expression toward in-groups and a decrease toward out-groups (Matsumoto et al., 2008a; Safdar et al., 2009). Developing the Display Rule Assessment Inventory, Matsumoto et al. (2005) reported that people often amplify emotions to friends and mask emotions to strangers, suggesting that it is more ‘proper’ to express intense emotions to intimates rather than to distant connections. Furthermore, display targets’ status also affects emotional behaviors in that individuals of higher status are privileged to express emotions and possess more freedom, whereas the emotional displays of individuals holding a lower status may be perceived as inappropriate (Cheshin, 2020). For example, Japanese individuals mask negative emotions when they express them to a higher-status experimenter in a negative situation (Matsumoto et al., 2008a) and are more receptive to expressing negative emotions toward lower-status individuals (Matsumoto, 1990). Moreover, research revealed that individuals alter their emotional expressions when they interact with the opposite sex. Particularly, individuals exhibited less negative expressive behavior while interacting with the opposite sex (Fischer et al., 2003).

As for social setting, despite its great scope, Japanese culture emphasizes the norms of expression in public and private. Public expressions correspond to “tatemae,” which refers to socially tuned motives or intentions. In contrast, private expressions correspond to “honne,” which represents individuals’ deep motives or intentions (Honna and Hoffer, 1986). In other words, the Japanese are expected to adjust or suppress expressions to fit their social climate in public, with real emotions hidden or masked to comply with norms. However, this display rule is not limited to Japan; Friedman and Miller-Herringer (1991) observed that people in other cultures also tended to conceal the happiness of triumph in public. Evers et al. (2005) also revealed that women expressed less anger in social settings than in anonymous ones. These results indicate that public settings could be more normative than private settings. Moreover, emotional behaviors are affected by the goals of interactions and the formality of the settings. For example, intense expressions are more appropriate for high-stake conflict resolution than in general service settings (Cheshin, 2020). Further, inhibited expressions are more likely to be requested in formal settings than in informal ones (Moran et al., 2013).

The intensity and manner of presentation also vary according to emotions. Wagner and Lee (1999) argued that display rules have a greater impact on negative emotions because revealing them can challenge self-presentation. For example, individuals tend to suppress sadness in the presence of others, since expressing sadness represents vulnerability and is, therefore, regarded as inappropriate (Jakobs et al., 2001). An early experimental study showed that the Japanese tend to display positive affect and reduce negative expressions more than do Americans when viewing stressful films in the presence of the experimenter (Friesen, 1972). Recent research additionally suggested that the Japanese are more likely to suppress socially disengaging emotions (Schouten et al., 2020). Further, even preschoolers were observed to mask disappointment with positive emotions when receiving unsatisfactory gifts (Ip et al., 2021). Upon investigating the Japanese expression management techniques, Inoue (2008) noted that negative emotions are more likely to be masked, but are rarely simulated. In contrast, happiness tends to be simulated to conceal negative emotions, but is rarely masked. Generally, positive emotions are expressed as much as they are experienced or even amplified, whereas negative emotions are more controlled and minimized, considering that negative expressions can be destructive to social relationships (Matsumoto et al., 2005; Safdar et al., 2009).

Additionally, cultural values promulgate a set of emotional ideologies that affect the extent of differentiation between emotional behaviors across contexts (Matsumoto et al., 2009), along with the types of emotion management (Matsumoto et al., 2008b). Individualism endorses higher expressivity norms in general, whereas collectivist cultures foster greater differentiation across social situations and encourage the adjustment of emotional responses to the contexts (Matsumoto et al., 2008a, 2009). Given the collectivist culture of Japan, in which the concept of self is interdependent on others (Markus and Kitayama, 1991), the norm of suppressing negative expressions to maintain a harmonious interpersonal atmosphere seems to be more prominent (Ekman and Friesen, 1975; Nakamura, 1991). Furthermore, under social norms discouraging the expression of intense emotions (Safdar et al., 2009), the Japanese are relatively emotionally restrained (Uchida et al., 2009; Dzokoto et al., 2018) and endorse greater emotion regulation (Wice et al., 2019).

### 2.2. The effects of social contexts on emoji use

Given the relative newness of emojis, research on the influence of social contexts on their use is much limited than that on facial expressions. While most existing studies did not aim to investigate emotion norms, much insight can be gained from existing research. First, inconsistent conclusions have been drawn regarding emoji use with different targets. In some studies, no difference was found in the frequency of emoticon (Xu et al., 2007) or emoji usage (Yamamoto and Kimura, 2017) when chatting with different partners. One explanation for this is that users want to convey their feelings better to those with low intimacy than to their friends (Xu et al., 2007). However, some studies have indicated that increased intimacy in a relationship is associated with more frequent and diverse use of emojis (Shi et al., 2019; Jones et al., 2020; Teh et al., 2020). Additionally, fewer emojis are used with elders and individuals in authority (Liu and Sun, 2020), and a changed pattern of emojis is found when individuals chat with those from a different gender (Wolf, 2000). These heterogeneous results could stem from different digital emotion norms across cultures or demographic characteristics. Hence, these findings should be explored further across different cultures to understand to what extent digital norms are a product of culture. Therefore, this study investigated whether emotional expression using emojis is adjusted to different targets in a Japanese cultural context.

Second, regarding social contexts, the effect of the social setting on emoticons has been reported; fewer emoticons are used in task-oriented contexts than in socio-emotional contexts (Yigit, 2005; Derks et al., 2007; Xu et al., 2007). Kaye et al. (2016) also found that individuals tend to decrease their use of emoticons in formal emails, considering their contextual demands. Similar to the use of facial displays, individuals prefer using emojis in private settings, such as in chats and text messaging, in contrast to public settings such as social media posts and group chats (Jones et al., 2020; Liu and Sun, 2020). However, unlike a real smile, smileys in a formal setting, such as work, can negatively impact first impressions (Glikson et al., 2018), indicating probable differences in emotion rules on facial displays and emojis. Additionally, women are particularly sensitive to the appropriateness of emojis in business settings and perceive leaders using emojis as less effective (Riordan and Glikson, 2020). However, despite the emphasis on the normality of public expression in the Japanese culture, there has been little relevant research on Japan. Therefore, this study investigated whether the use of emojis to express emotions differs between public and private settings.

Third, in terms of emotional aspects, emojis are generally used to express positive emotions (Vidal et al., 2016). The emotional value of emojis and contexts are also consistent, which means that positive (negative) emojis are more often used in positive (negative) contexts (Derks et al., 2007). While individuals are more emotionally expressive in positive contexts than in negative contexts during FTF interactions, studies have found no difference in the frequency of emojis used in positive and negative situations (Derks et al., 2007; Hancock et al., 2007). These divergent findings could be reasoned by the different emotion rules for offline and online behaviors. However, they are more likely because the frequency of emoji use does not necessarily reflect the degree of emotional expression. Indeed, individuals do not often, if at all, use emojis when their expressions are intense (Kato et al., 2009). It has also been argued that the association between emojis and the intensity of expression varies by the expressed emotions and individuals (Shoeb and de Melo, 2020). Further, emojis that represent similar emotions, such as 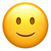 and 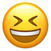, which are used to display happiness, can also be entirely different in their emotional intensity (Krekhov et al., 2022). Thus, even though emojis are used with the same frequency, the intensity and type of expressions in positive and negative contexts may differ. Similarly, the more frequent use of emojis with close individuals or in private situations does not always indicate a greater intensity of an expression. However, previous studies failed to consider this and examined only the variation in the frequency of emojis used across contexts. The current study filled this gap by investigating the degree of emotional expression and the type of emojis used in different contexts. Indeed, asking participants to select emoticons from a designed list (Derks et al., 2007) or self-reporting the frequency of emoticons used (Kaye et al., 2016) can be very different from the daily use of emoticons Appropriate changes were made to the method by asking participants to type emojis as they normally use them. This modulation allows a detailed exploration of emojis used in a given situation that has hardly been performed before.

Overall, the present study aimed to investigate how emotional expressions and the use of emojis are adjusted to different targets, public or private settings, and positive or negative contexts. Since emojis have been found to be as emotionally intense as facial displays (Fischer and Herbert, 2021), with similar behavior patterns found in both, the present study anticipated that emotional expressions with emojis would be adjusted to the social contexts similar to those with facial expressions. Particularly, four hypotheses were proposed: (**H1)** Individuals disclose more emotions with emojis when they are closely related to usage targets. (**H2)** Individuals express more emotions with emojis in private than in public settings. **(H3-1)** Individuals express more emotions with emojis in positive contexts than in negative contexts. Consistent with previous findings (Derks et al., 2007), it was expected that **(H3-2)** individuals use more positive emojis in positive contexts and more negative emojis in negative contexts.

### 2.3. Emotion management and psychological well-being

Many empirical studies have revealed that emotional expressions have many benefits and are associated with improved mental health. For example, individuals who are generally emotionally expressive are more likely to be liked (Collins and Miller, 1994) and benefit from satisfactory relationships (Morry, 2005). Emotional expression allows more opportunities for social support (Pollastri et al., 2018) and promotes increased connections with others after the displayers receive responses from listeners (Rimé, 2007, as cited in Pollastri et al. 2018). With supportive relationships and social bonds, disclosing emotions may potentially contribute to well-being, which is validated by many experimental results reporting a positive correlation between emotional expression and increased psychological well-being (Pennebaker et al., 2001; Gable et al., 2004).

By contrast, although emotion management is suggested to be associated with accomplishment in certain cases, it is usually accompanied by many unhealthy outcomes. Ekman (1970) proposed four emotion management techniques to manage emotional expressions to be consistent with display rules: de-intensifying, over-intensifying, or masking the felt emotions and attempting to be neutral. Hochschild (1983) argued that in addition to the aforementioned manipulation of surface behaviors (surface acting), people also attempt to modify internal feelings (deep acting) to bring emotional displays in line with emotion rules. Through meta-analysis drawing upon multiple studies, research indicates that surface acting is usually related to impaired psychological well-being (Hülsheger et al., 2010; Mesmer-Magnus et al., 2012), such as depressive symptoms (Kim and Choo, 2017), increased emotional exhaustion (Zhang et al., 2018), and elevated negative affect (Judge et al., 2009). However, deep acting is less harmful to mental health (Grandey, 2003; Hülsheger et al., 2010), suggesting that discordant emotion management between felt and displayed emotions is more likely to threaten mental health (Mesmer-Magnus et al., 2012; Lee et al., 2016). When people subordinate genuine feelings to social expectations, uncomfortable emotional dissonance arises, which generates discrepancies between the inner experience and what one is supposed to feel (Carminati, 2021). Moreover, the demands of dealing with negative emotions and being sensitive to others’ emotions are positively associated with emotional exhaustion (Zapf, 2002). However, it is noteworthy that the results of emotion management are culturally different. For example, emotional suppression is more prevalent in Eastern countries, especially in collectivist cultures, with less deleterious mental health outcomes reported than in Western cultures (Matsumoto et al., 2008a; Cheung and Park, 2010; Schouten et al., 2020). This may be because people in cultural environments that endorse emotion management strategies suppress emotions in situations that they perceive as beneficial, which may lead to less harmful outcomes (Schouten et al., 2020).

Empirical results have shown associaions between mental health with the frequency and diversity of emoji use (Vuillier et al., 2018; Callender et al., 2022). Emojis are also reported to be able to provide a new method for assessing mental health (Van Dam et al., 2019) and detecting burnout in remote work (Lu et al., 2022). Even though prior findings suggest that emoji use and psychological well-being are closely related, little research has focused on the psychological impact of emotion management using emojis. In light of the above reasons, this study investigated the relationship between emotional expression, emotion management, and mental health based on emoji use. The final hypothesis was proposed as follows: **(H4)** Emotional expressions with emojis would be positively associated with well-being; however, given the collectivistic cultural background of Japan, emotion management with emojis would be weakly and negatively associated with well-being. As previously stated, emotion management refers to the adjustment and modification of emotional expressions under the constrains of display rules. Accordingly, emotion management with emojis is defined as using emojis to tune and modulate emotional reactions (e.g., expression intensity and choice of emojis) according to the contexts, in alignment with the display rules.

## 3. Methods

### 3.1. Participants

The survey was conducted nationwide in Japan with participants aged 10–29 years, since individuals of this age group represent the largest segment of digital communication users (Ministry of Internal Affairs and Communications of Japan, 2021). With the assistance of the corporation holding the most downloaded Japanese keyboard application, “Simeji,” the present study published an online questionnaire survey on its official website. This target population was chosen because the application is frequently used by young generations in their 10s and 20s in Japan. With the function of changing backgrounds and providing predictive emojis according to the typed texts, “Simeji” has a high popularity among the Z generation, especially among females, as it responds to the culture of young Japanese people (MarkeZine, 2016). In total, 1,289 participants were included in this study, comprising 1,211 females and 78 males. Among them, 1,085 participants were adolescents (aged 12–18) and 204 participants were adults (aged 19–29). The mean age of the participants was 15.9 years (SD = 3.4).

### 3.2. Procedures

The present study is part of a larger research on the use of emojis in Japan, with the survey being pre-tested to ensure the validity of the survey instrument and its measurements (Ruel et al., 2016). Before answering the questionnaire, the participants were informed that they could voluntarily participate in the study, refuse to answer the questionnaire, and interrupt their responses at any time. Participants provided demographic data and rated their frequency of emoji usage on a 6-point scale (1 = rarely used; 2 = 1–3 times a week; 3 = 4–6 times a week; 4 = 1–3 times a day; 5 = 4–6 times a day; and 6 = more than 7 times per day). They were presented with 12 short Internet chats that varied in the social context and were asked to imagine online interactions based on the prompted contexts. They then responded to the chats with emojis (or without emojis) as they would do in daily communications and answered about the intensity to which they expressed emotions through the emojis. In this procedure, participants could input any emoji and input “0” if they did not want to use any emoji. The expression intensity was rated on a 5-point scale (Ekman and Friesen, 1978), ranging from 0–20% to 80–100%. Finally, they answered questions related to the measurement of their mental health status.

### 3.3. Materials

#### 3.3.1. Internet chats

Based on the proposed hypotheses, the chat contexts varied in (H1) usage targets (same-sex friend, opposite-sex friend, someone of higher social status, someone unfamiliar), (H2) private chats or public chats, and (H3) the valence of the social context (positive vs. negative) (see Table 1). The four usage targets were designated because the Japanese regard same-sex friends as being closer than friends of the opposite sex (Hiroya, 2007). Further, Japanese social norms expect individuals to treat people with higher social status, and those that are unfamiliar, with consideration and respect (Kato et al., 2005; Yamamoto and Kimura, 2017). Internet chats were designated with texts for which participants could input both positive and negative emojis to decrease the influence of the textual message on emoji selection. The contents of these chats are also related to the daily lives of young Japanese individuals.

**Table 1:** Internet chats and hypotheses.

No.	Chat condition		Hypotheses
Q1	Context 1 (interactor won a contest)	Same-sex friend	Individuals disclose more emotions with emojis when they are closely related to usage targets (H1)
Q2		Opposite-sex friend	
Q3		Higher social status	
Q4		Someone unfamiliar	
Q5	Context 2 (interactor broke a promise)	Same-sex friend	
Q6		Opposite-sex friend	
Q7		Higher social status	
Q8		Someone unfamiliar	
Q9	Positive context (interactor won a context)	Private chat	Individuals express more emotions in private than in public (H2) and in positive than in negative contexts (H3)
Q10		Public chat	
Q11	Negative context (Unsatisfied with the interactor)	Private chat	
Q12		Public chat	

**Figure 1:**
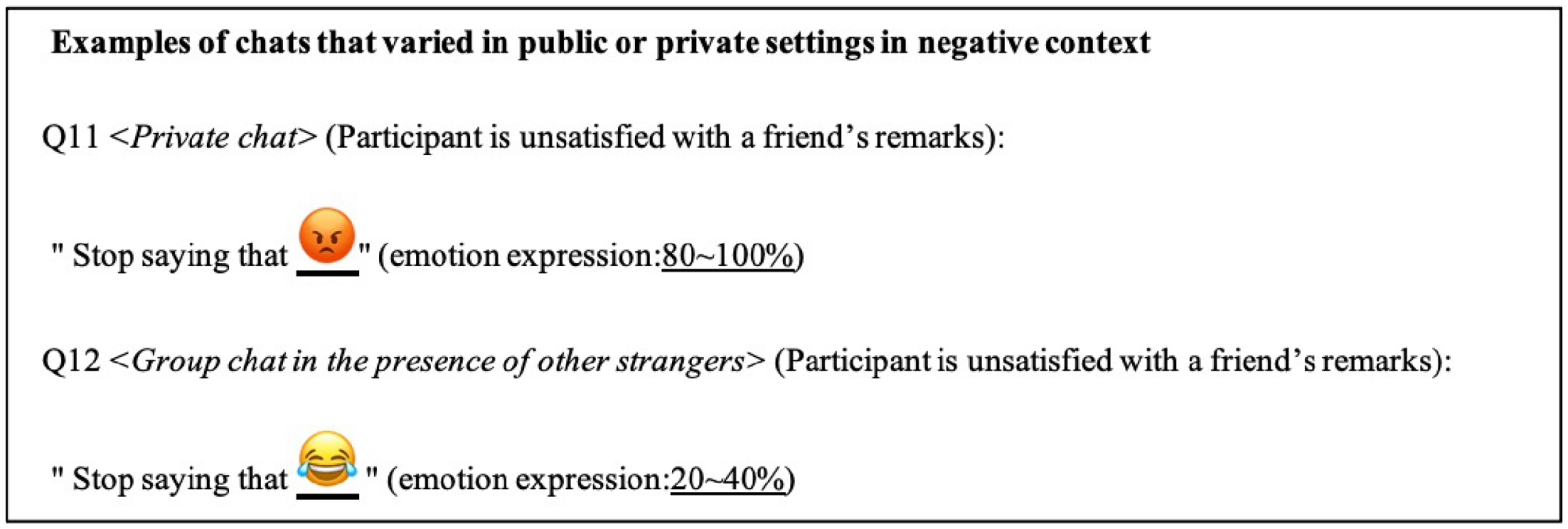
Example answers of Internet chats.

Chats Q1–Q8 were designed to investigate the influence of usage targets on emotional expression using emojis (H1), which varied among chat partners and contexts. In the first context, different interactors had won a contest and the participant was asked whether they had heard the news, wherein the participant was not interested. In the second context, the participant was angry with the interactors who had broken a promise. Chats Q9–Q12 were designed to investigate how emotional expressions using emojis are adjusted to public or private settings (H2) and the valence of context (H3). In a positive context, the participant congratulated a friend on winning a contest. In the negative context, the participant was angry with the friend’s remarks. Particularly, private chat referred to a situation wherein the participant interacted with a friend, whereas group chat was relatively public, involving other strangers. Examples of responses in private and group chats under negative context are illustrated in Figure 1. All of the Internet chats are presented in Supplementary Text S1.

#### 3.3.2. Measures

Two measures of psychological well-being were considered to achieve the aims of this study.

##### 3.3.2.1. Depressive symptoms

Prior research has pointed to associations between emotion management with depressive symptoms (Kim and Choo, 2017) and the risk of depression (Chun et al., 2020). Therefore, the Center for Epidemiological Studies Depression Scale (CES-D), which is a common measure of depressive symptoms, was used (Calvo and Peters, 2014). The present study was included in a larger survey on emoji use; to reduce the burden on the participants, three items with the highest contribution rates from the factor analysis of the CES-D scale for Japanese female university students were applied in the questionnaire (Sugiura and Umaoka, 2003). Participants were asked to respond on a 5-point scale ranging from 1 (not at all) to 5 (5 days or more in a week). Cronbach’s α for depressive symptoms was 0.81.

##### 3.3.2.2. Subjective happiness

The Japanese Cabinet Office (2012) used subjective happiness as a scale to capture the subjective well-being of Japanese individuals. Thus, this scale was included in the present study, and respondents had to rate their subjective well-being on a range of 1 (very unhappy) to 10 (happy).

### 3.4. Data analysis and emoji coding

The present study used IBM SPSS Statistics 25 for data analysis and SAS 9.4 mainly for data processing. First, an analysis of variance (ANOVA) was performed to examine whether emotional expression differed by targets and contexts (H1–H3-1). Subsequently, multiple comparisons were conducted using the Bonferroni method. Here, the degree of emotional expression was counted as the midpoint of each quintile (e.g., 0–20% was counted as 10%, and 80–100% as 90%). Second, to examine the emotional valence of emojis used in different situations (H3-2), a residual analysis was performed to determine the likelihood of a particular emoji category to be used. Scholars recommended residual analysis to further investigate a statistically significant chi-square test result (Agresti, 2002; Sharpe, 2015). A residual represents the difference between the observed and expected values for a cell. The larger the residual, the greater the contribution of the cell to the obtained chi-square value. Adjusted residuals are calculated by dividing the raw residual by the square root of the expected value as an estimate of the raw residual’s standard deviation, which is also provided by SPSS (Sharpe, 2015). Usually, an adjusted residual exceeding ±1.96 indicates that the number of cases in that cell is significantly larger or smaller than expected, with a significance level of 0.05 (IBM, 2020; Nomura et al., 2022). Finally, multiple linear regressions were performed to examine whether emotional expression and management using emojis were associated with depressive symptoms and subjective happiness (H4). Since prior studies reported an association between the frequency of emoji use and mental health, the present study included frequency of emoji use, gender, and age as control variables in the regressions. The two regressions had no multicollinearity through variance inflation factor, with a maximum value of 1.03.

Table 2 presents the descriptive statistics and correlations for each related variable. Since emotional intensity refers to the strength of emotional response (Larsen and Diener, 1987), the sum of expression intensity answered in 12 chats was used as the variable of emotional expression using emojis. Additionally, the sum of the absolute values of variations in expression intensities adjusted to different contexts and targets was used as the variable of emotion management. To elaborate, it is the sum of the absolute differences between expression intensity in private and public settings (|Q9-Q10| + |Q11-Q12|), positive and negative contexts (|Q9-Q11| + |Q10-Q12|), and toward same-sex friends versus the other three targets (|Q2-Q1| + |Q3-Q1| + |Q4-Q1|plus|Q6-Q5| + |Q7-Q5| + |Q8-Q5|). The calculation on targets was based on same-sex friends since prior findings showed that same-sex friends are the closest of the four targets, with whom individuals are the most expressive (Hiroya, 2007; Safdar et al., 2009). Table 2 illustrates that emoji frequency was significantly and positively correlated with both emotional expression and management. Age was significantly and positively correlated with emoji frequency and emotional expression, whereas it was negatively correlated with emotion management.

**Table 2:** Descriptive statistics and inter-correlations among variables.

	M (SD)	Range	1	2	3	4	5
1. Age	15.93 (3.40)	(12–29)	1				
2. Emoji frequency	4.60 (1.73)	(1–6)	0.06^†^	1			
3. Emotion expression	5.75 (2.02)	(1.2–10.8)	0.07**	0.15**	1		
4. Emotion management	2.50 (1.19)	(0–6)	−0.06*	0.07*	0.03	1	
5. Depressive symptoms	9.35 (3.39)	(3–15)	−0.02	−0.07*	−0.08**	0.05†	1
6. Subjective happiness	6.45 (2.36)	(1–10)	−0.02	0.13**	0.17**	0.01	−0.48**

M, mean; SD, standard deviation. †*p* < 0.1; *p < 0.05; **p < 0.01.

All emojis in the chats were coded and categorized on the basis of the Unicode Standard (Version 12.1). This is because Unicode provides a detailed classification of all emojis and is a universal character-encoding standard for written characters. Since research on the use of emojis is limited in Japan, this study adopted a detailed classification to prevent some special categories of emoji use from going undetected. Furthermore, although participants were asked to answer using emojis instead of emoticons and the differences between them were explained, answers using emoticons were still observed. To this end, “no emoji” (do not use emojis) and “emoticons” were added to the categories. All emoji categories are presented in Supplementary Table S1.

## 4. Results

### 4.1. Emotional expression of emojis with different targets (H1)

**Figure 2:**
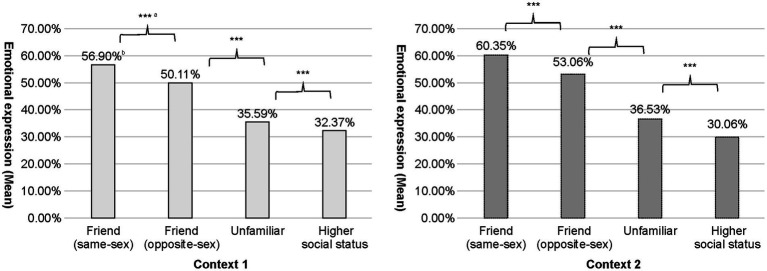
Emotional expression (mean) with different relationships (***p < 0.001). ^a^Significant differences were found in multiple comparisons (all *p* < 0.001). ^b^The mean was based on the midpoint of each quintile (e.g., 10% for 0–20%).

A 2 (context) × 4 (usage target) ANOVA was conducted on emotional expressions using emojis to test the first hypothesis. The main effect of the usage target was *F*(2, 3175) =667.44, *p <* 0.001, *η^2^p* = 0.34 Furthermore, multiple comparisons were conducted using Bonferroni’s method. The results showed a significant difference in emotional expression between each pair of targets in both contexts (*p <* 0.001). As shown in Figure 2, the intensity of emotional expression decreased in the order of same-sex friends, opposite-sex friends, someone unfamiliar, and individuals with a higher social status in each context.

The most frequently used categories of emojis with different targets are summarized in Tables 3, 4. Chi-square tests revealed the significant associations between the emoji categories and the targets in both contexts (all *p <* 0.001). The adjusted residuals (AR) were thereafter calculated to determine which emoji category contributes to the results. AR above ±1.96 indicates that a particular category is used significantly more or less than expected. In the first context, the participants heard that their partner had won a contest, but they were not interested. Table 3 indicates that more individuals chose not to use emojis with individuals of higher social status (28.7%) or those they were unfamiliar with (24.7%), in contrast to same-sex (13.0%) and opposite-sex friends (12.0%). Regardless of the recipient, the two most used emoji categories were face-smiling and face-concerned. Residual analysis showed that “wacky” emojis were more likely to be used with same-sex friends, such as the face-tongue category (e.g., 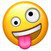; AR = 3.5) and monkey-face category (e.g., 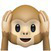; AR = 2.7). Emojis that euphemistically express emotions, such as the person-gesture category (e.g., 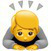), were used primarily with those of higher social status (AR = 7.1), and rarely with same-sex (AR = −2.1) and opposite-sex friends (AR = −3.8). In the second context, individuals were asked to express their anger when their partner broke a promise. Table 4 illustrates that, compared with the same-sex (13.8%) and opposite-sex friends (15.8%), emojis were not used with individuals they were unfamiliar with (25.5%) or those with higher social status (29.8%). Furthermore, residual analysis showed that the face-negative category (e.g., 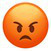) that directly expressed negative emotions was used more with same-sex (AR = 8.0) and opposite-sex friends (AR = 9.1), but rarely with unfamiliar individuals (AR = −6.0) and those of higher status (AR = −10.5). Additionally, the person-gesture category (e.g., 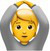) were frequently used with high-status individuals (AR = 4.9).

**Table 3:** Most used emoji categories with different targets (interactor won a contest).

Rank	Friend (same-sex)			Friend (opposite-sex)			Unfamiliar			Higher social status		
	Example	Category	Percentage (accum.)	Example	Category	Percentage (accum.)	Example	Category	Percentage (accum.)	Example	Category	Percentage (accum.)
1	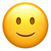	Face-smiling	16.5 (16.5)	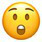	Face-smiling	17.7 (17.7)	/	No emoji	24.7 (24.7)	/	No emoji	28.7 (28.7)
2	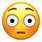	Face-concerned	16.0 (32.5)	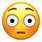	Face-concerned	16.9 (34.6)	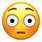	Face-concerned	17.9 (42.6)	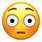	Face-concerned	20.3 (49.0)
3	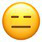	Face-neutral-skeptical	15.2 (47.7)	/	No emoji	14.9 (49.5)	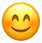	Face-smiling	16.5 (59.0)	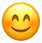	Face-smiling	16.2 (65.2)
4	/	No emoji	13.0 (60.7)	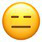	Face-neutral-skeptical	12.0 (61.4)	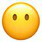	Face-neutral-skeptical	8.0(67.0)	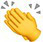	Hands	5.9 (71.1)
5	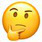	Face-hand	12.2 (72.8)	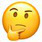	Face-hand	10.1 (71.5)	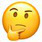	Face-hand	7.1 (74.1)	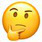	Face-hand	5.1 (76.2)
6	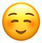	Face-affection	3.9 (76.8)	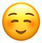	Face-affection	3.7 (75.3)	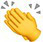	Hands	2.9 (77.1)	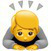	Person-gesture	4.7 (80.9)
7	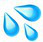	Emotion	2.8 (79.6)	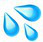	Emotion	2.7 (78.0)	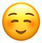	Face-affection	2.8 (79.8)	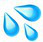	Emotion	3.4 (84.3)
8	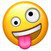	Face-tongue	1.9 (81.6)	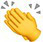	Hands	2.2 (80.2)	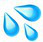	Emotion	2.6 (82.5)	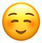	Face-affection	2.7 (87.0)
9	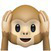	Monkey-face	1.8 (83.4)	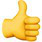	Hand-fingers-closed	2.1 (82.3)	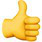	Hand-fingers-closed	2.4 (84.8)	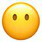	Face-neutral-skeptical	2.7 (89.6)
10	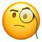	Face-glasses	1.5 (84.9)	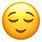	Face-sleepy	1.7 (84.0)	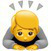	Person-gesture	1.8 (86.6)	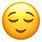	Face-sleepy	1.8 (91.4)

**Table 4:** Most used emoji categories with different targets (interactor broke a promise).

Rank	Friend (same-sex)			Friend (opposite-sex)			Unfamiliar			Higher social status		
	Example	Category	Percentage (accum.)	Example	Category	Percentage (accum.)	Example	Category	Percentage (accum.)	Example	Category	Percentage (accum.)
1	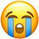	Face-concerned	30.5 (30.5)	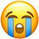	Face-concerned	30.7 (30.7)	/	No emoji	25.5 (25.5)	/	No emoji	29.8 (29.8)
2	/	No emoji	13.8 (44.3)	/	No emoji	15.8 (46.5)	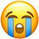	Face-concerned	25.0 (50.5)	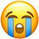	Face-concerned	25.8 (55.6)
3	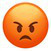	Face-negative	13.0 (57.2)	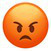	Face-negative	12.0 (58.5)	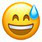	Face-smiling	15.3 (65.8)	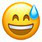	Face-smiling	15.9 (71.5)
4	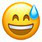	Face-smiling	11.6 (68.9)	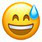	Face-smiling	10.6 (69.1)	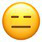	Face-neutral-skeptical	6.9 (72.7)	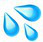	Emotion	3.9 (75.4)
5	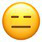	Face-neutral-skeptical	10.4 (79.2)	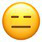	Face-neutral-skeptical	9.6 (78.7)	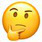	Face-hand	3.7 (76.4)	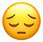	Face-sleepy	2.9 (78.3)
6	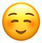	Face-affection	2.4 (81.6)	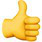	Hand-fingers-closed	2.8 (81.5)	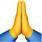	Hands	3.1 (79.5)	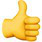	Hand-fingers-closed	2.7 (80.9)
7	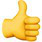	Hand-fingers-closed	2.3 (83.9)	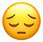	Face-sleepy	2.7 (84.2)	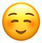	Face-affection	2.9 (82.4)	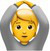	Person-gesture	2.5 (83.4)
8	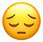	Face-sleepy	2.1 (86.0)	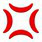	Emotion	2.5 (86.7)	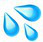	Emotion	2.8 (85.2)	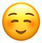	Face-affection	2.4 (85.9)
9	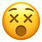	Face-unwell	1.9 (88.0)	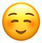	Face-affection	2.0 (88.7)	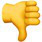	Hand-fingers-closed	2.4 (87.6)	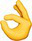	Hand-fingers-partial	2.3 (88.2)
10	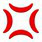	Emotion	1.6 (89.6)	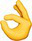	Hand-fingers-partial	1.3 (89.9)	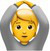	Person-gesture	1.6 (89.2)	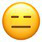	Face-neutral-skeptical	1.7 (89.9)

### 4.2. Emotional expression of emojis in public and private (H2)

To assess the difference between the emotional expressions of emojis in public and private settings, a 2 (valence of context) × 2 (private or public) ANOVA on the extent of emotional expressions was conducted. A significant main effect of private or public context, *F*(1,1260) =1496.24, *p <* 0.001, *η^2^p* = 0.54, was found. However, this main effect was qualified by a significant interaction between context valence and private or public context on the use of emojis for emotional expressions, *F*(1, 1260) =179.86, *p <* 0.001, *η^2^p* = 0.13. Bonferroni-adjusted *post hoc* test indicated that, during positive disclosures, the intensity of emotional expressions in private was significantly more than that in public (M_diff_ = 0.27, *p <* 0.001, 95% CI of the difference = 0.26 to 0.29). In the negative context, a decreased difference between expression intensity in private and public was found (M_diff_ = 0.14, *p <* 0.001, 95% CI of the difference = 0.13 to 0.16), indicating a stronger effect of settings in the positive context.

### 4.3. Emotional expression of emojis in different context valences (H3)

**Figure 3:**
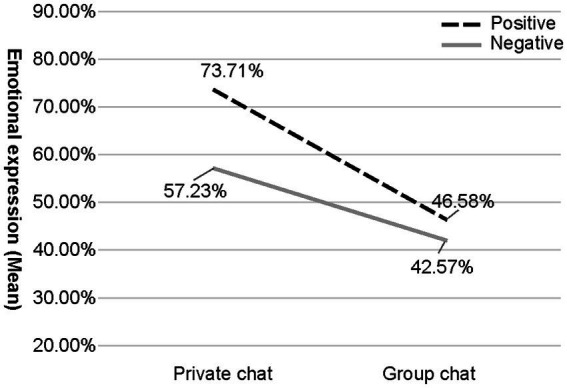
Emotional expression (mean) in different social contexts. The mean was based on the midpoint of each quintile (e.g., 10% for 0–20%).

A 2 (valence of context) × 2 (private or public) ANOVA on emotional expression using emojis was conducted to test Hypothesis H3-1. There was a significant effect of context valence, *F*(1,1260) =270.25, *p <* 0.001, *η^2^p* = 0.18 However, this main effect of context valence was also qualified by the significant interaction stated above. According to the *post hoc* test using Bonferroni’s method, individuals expressed significantly more emotions using emojis in positive contexts than in negative contexts in both private (M_diff_ = 0.16, *p <* 0.001, 95% CI of the difference = 0.15 to 0.18) and group settings (M_diff_ = 0.04, *p <* 0.001, 95% CI of the difference = 0.03 to 0.06), with a lower difference in the group setting, suggesting a stronger effect of context valence in private. Additionally, as shown in Figure 3, individuals used emojis to express the highest extent of emotion in private and positive contexts, whereas they expressed the lowest extent of emotion in public and negative contexts.

To test hypothesis H3-2 (individuals use more positive emojis in positive situations and more negative emojis in negative situations), first, a chi-square test was conducted for emotional expressions and emoji categories particularly because the intensity of expressions can influence the choice of emojis. Consequently, significant associations were found between emoji categories and expression intensity in all four cases (positive*private/public; negative*private/public; all *p <* 0.001). Thereafter, residual analyses were performed to determine the observations contributing to the significant differences. Given that emotional expression is a 5-point-scaled variable, which can lead to a large amount of data, only the significant results found at the lowest (0–20%) and highest expression intensity (80–100%) were summarized in Table 5 (positive contexts) and Table 6 (negative contexts). In the context of the present study, an adjusted residual exceeding ±1.96 indicates that a particular emoji category was more or less likely to be used than the expected values. Since Delucchi (1993), as cited in Sharpe (2015), suggested identifying those cells with the largest residuals, results were sorted by the absolute values. Whether the results were found in a public or a private context was listed in parentheses. The smiling, negative, and neutral face categories were bolded for clarity.

**Table 5:** Residual analysis of emoji categories and emotional expression in positive contexts.

	High expression intensity (80 ~ 100%)	Adjusted residual	Low expression intensity (0 ~ 20%)	Adjusted residual
Adjusted residuals>1.96	Person-gesture (public)	2.9**	No emoji (private)	15.3**
	Person-fantasy (public)	2.9**	No emoji (public)	13.1**
	Animal-marine (public)	2.9**	Hand-fingers-partial (private)	2.6**
	Face-hat (private)	2.3**	Warning (public)	2.1*
			Person-sport (public)	2.1*
			Person-role (public)	2.1*
			Face-tongue (public)	2.1*
			Face-costume (public)	2.1*
			Food-sweet (private)	2.0**
Adjusted residuals < −1.96	No emoji (private)	−5.8**	**Face-smiling (public)**	−4.4**
	Emoticons (private)	−2.1*	Hands (private)	−3.4**
	Sky & weather (private)	−2.0**	Event (private)	−2.9**
			Face-affection (public)	−2.8**
			Hands (public)	−2.3*
			Hand-fingers-closed (public)	−2.3*
			Face-concerned (private)	−2.2*

*p < 0.05; **p < 0.01; (*p* < 0.01 for adjusted residual > |2.58| and *p* < 0.05 for adjusted residual > |1.96|).

Table 5 shows that when individuals expressed intense emotions in positive contexts, they tended to use more emojis and fewer emoticons in private chats, and preferred person-gesture as well as person-fantasy categories in group chats. Conversely, when the intensity of expression was diminished in a positive context, individuals tended not to use emojis and especially decreased the use of smiling faces in group chats. Table 6 shows that when emotions were strongly expressed in negative contexts, participants tended to avoid using emojis and smiling faces and were more inclined to use negative emojis in group chats. However, when participants attenuated their negative expressions, they avoided using emojis and neutral faces and preferred to send smiling faces instead. Moreover, the use of negative emojis decreased in private chats.

**Table 6:** Residual analysis of emoji categories and emotional expression in negative contexts.

	High expression intensity (80 ~ 100%)	Adjusted residual	Low expression intensity (0 ~ 20%)	Adjusted residual
Adjusted residuals > 1.96	Hand-fingers-closed (public)	5.2**	No emoji (private)	7.6**
	No emoji (private)	5.1**	No emoji (public)	6.8**
	**Face-negative (public)**	3.3**	**Face-smiling (public)**	3.9**
	No emoji (public)	3.3**	Face-tongue (private)	2.7**
	Hand-single-finger (public)	3.0**	Face-glasses (private)	2.4*
	Face-costume (public)	3.0**	Animal-mammal (private)	2.4*
	Hand-single-finger (private)	3.0**	Face-tongue (public)	2.0**
	Monkey-face (public)	2.8**	**Face-smiling (private)**	2.0*
	Person-symbol (public)	2.8**		
	Dishware (public)	2.8**		
	Event (public)	2.8**		
Adjusted Residuals < −1.96	**Face-smiling (public)**	−4.0**	**Face-neutral-skeptical (public)**	−4.1**
	Face-concerned (private)	−3.3**	Face-concerned (public)	−3.5**
	**Face-smiling (private)**	−2.9**	**Face-negative (private)**	−3.5**
	Hands (private)	−2.3*	**Face-neutral-skeptical (private)**	−2.3*
			Face-concerned (private)	−2.1*

*p < 0.05; **p < 0.01; (*p* < 0.01 for adjusted residual > |2.58 | and *p* < 0.05 for adjusted residual > |1.96|).

### 4.4. Emotion management with emojis and well-being (H4)

Multiple regression analyses were used to ascertain the relationship between well-being, emotional expression, and emotion management in emoji use (H4). When the score of depressive symptoms was included as the dependent variable, a significant regression equation was found (*F* (5,1249) =4.68, *p <* 0.001) with an R^2^ of 0.02. The results (see Table 7) showed that emotional expression with emojis negatively predicted depressive symptoms (*β* = −0.07, *t* = −2.50, *p* = 0.01), whereas emotion management with emojis positively predicted depressive symptoms (*β* = 0.05, *t* = 1.84, *p* = 0.07). Furthermore, the results revealed that emoji usage frequency negatively predicted depressive symptoms (*β* = −0.05, *t* = −1.87, *p* = 0.06). The regression model was also significant when subjective happiness was included as the dependent variable (*F*(5,1249) =11.50, *p* < 0.001, R^2^ = 0.04). Consequently, the results (see Table 7) indicated that emotional expression with emojis positively predicted subjective happiness (*β* = 0.16, *t* = 5.74, *p* < 0.001). However, no significant association was found between emotion management and subjective happiness. Additionally, those who frequently used emojis (*β* = 0.10, *t* = 3.73, *p* < 0.001) were more likely to report high scores for subjective happiness. No significant associations were found between age, depressive symptoms, and subjective happiness.

**Table 7:** Regression analyses for mental health.

Predictor	Depressive symptoms			95% CI for B		Subjective happiness			95% CI for B	
	*B*	β	SE	Low	Up	*B*	β	SE	Low	Up
Age	−0.01	−0.01	0.03	−0.06	0.05	−0.02	−0.03	0.02	−0.06	0.02
Gender (*F* = 1, M = 0)	1.26	0.09**	0.40	0.47	2.06	−0.63	−0.06†	0.28	−1.18	−0.09
Emoji frequency	−0.10	−0.05†	0.06	−0.21	0.01	0.14	0.10***	0.04	0.07	0.22
Emotional expression	−0.12	−0.07*	0.05	−0.21	−0.03	0.19	0.16***	0.03	0.12	0.25
Emotion management	0.15	0.05†	0.08	−0.01	0.31	−0.001	−0.001	0.05	−0.11	0.11
*R*^2^ (adjusted *R*^2^)	0.02(0.02)					0.04(0.04)				
*F* value	4.68***					11.50***				

B, regression weight; β, standardized regression weight; SE, standardized error of the regression weight. †*p* < 0.1; *p < 0.05; **p < 0.01; ***p < 0.001.

## 5. Discussion

The present study aimed to investigate (1) whether emotional expressions using emojis are adjusted to social contexts consistently with facial displays and (2) how emotion management with emojis is related to psychological well-being. Although previous studies have provided insights into the contextual demands on emoji use, few have aimed to investigate the emotion norms involved. Furthermore, existing studies explored only the frequency of emoji usage while ignoring the fact that the extent of emotional expression can vary when the same number of emojis are used. This study fills this gap by examining how emotional expressions are adapted to different targets and contexts. Moreover, the variance of emoji types was analyzed according to each context while the intensity of expressions differed. Based on current knowledge, the present study is also the first to investigate how emotion management and expression with emojis are related to mental health.

The first hypothesis proposed that individuals would use emojis to express more emotions to closely related individuals, similar to FTF communication. ANOVA showed a significant main effect of targets, with participants expressing most emotions toward same-sex friends, followed by opposite-sex friends, unfamiliar individuals, and those of high status. Safdar et al. (2009) reported that the Japanese consider high-status people as a distant group, unfamiliar students as the middle group, and friends as the close group, with perceived closeness increasing in that order. Notably, although people are more expressive with their intimate counterparts, Japanese individuals significantly differentiate their expression with close and distant groups than people from other countries (Safdar et al., 2009). Moreover, Japanese people perceive same-sex friends as being more intimate than opposite-sex friends and are more expressive in same-sex friendships (Kito, 2005; Hiroya, 2007). These findings could explain why the participants used emojis for expressing more to same-sex friends, followed by the other three targets. Overall, the present results suggest that even in the digital world, people are more expressive in intimate relationships, which supports the first hypothesis. Additionally, the current results are consistent with those of Jones et al. (2020) and Liu and Sun (2020), while they contradict those of Xu et al. (2007) and Yamamoto and Kimura (2017). This is perhaps because most participants in Xu et al. (2007) frequently used emoticons, which might have led to frequent use with any target. Yamamoto and Kimura (2017) asked participants to converse about their college life. However, the limited opportunity for emotional expressions on this topic might have resulted in the lack of difference in the frequency of emoji use between friends and strangers.

In addition to the extent of emotional expression, individuals used different types of emojis that were adjusted to targets. Generally, the current study replicated prior findings that individuals use fewer emojis with those who are distantly related (Jones et al., 2020). Furthermore, the results suggested that participants used “wacky” and “anger” emojis with their same-sex friends, while they used euphemistic expressions of emojis with less intimate individuals. Combined with the above results, the present study suggests that individuals may selectively reduce the degree of emotional expression with non-intimates and choose emojis that indirectly express emotions. These pieces of evidence also support the idea that individuals are aware of the demands of the social context (Kaye et al., 2016) and consider social acceptance and appropriateness when using emojis. Additionally, emojis such as “
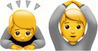
” were frequently used with more distantly related targets. In Japan, individuals use honorifics and polite words with strangers or those in positions of higher authority. Research has also argued that the Japanese approach to electronic communication is consistent with their inherent style of communication (Harada, 2004). Those emojis can thus be interpreted as the electronic equivalent of Japanese honorifics to show respect.

The second hypothesis predicted that individuals would express their emotions with emojis more in private settings than in public ones. Consequently, this hypothesis was supported by the ANOVA test showing a significant main effect of setting, with emotions expressed more in private chats than in public ones. Previous studies have also reported a higher frequency of emoji use in private chats than in group chats (Liu and Sun, 2020) and more in private messages in contrast to public social media posts (Jones et al., 2020). Individuals perceive social context cues and adjust their emotional behaviors to their definitions and interpretations of the contexts. When social situation cues are strong, individuals tend to be other-focused (Sproull and Kiesler, 1986). Similarly, emotional expressions with emojis may also be affected by online social contexts—individuals are more expressive in a relatively private context, while in public they turn their attention to others and care more about the appropriateness of emotional expression.

Hypothesis H3-1 was also supported by the ANOVA test, demonstrating a significant effect of contextual values, with emotions expressed more in positive than in negative contexts. For facial expressions, negative emotions are more likely to be suppressed because they are regarded as inappropriate (Safdar et al., 2009). Similarly, the expression of negative emotions, which have been considered destructive to interpersonal relationships, also decreased in the use of emojis. However, Derks et al. (2007) showed the lack of difference in the frequency of emoji use between positive and negative contexts, probably because emojis vary in their functions in different contexts. Emojis are used in positive contexts to increase the effectiveness of expressions (Takehara and Sato, 2004), while in negative contexts they are used to avoid embarrassment or to lighten the mood (Arakawa and Suzuki, 2004). Thus, even if emojis are used at the same frequency, their degree of expression may differ. Cultural differences in the display rules may be another reason for this. Experiments have shown that the Japanese are less likely to express negative emotions than Americans (Ekman and Friesen, 1975). Similarly, individuals from Asian cultural backgrounds are also less receptive to expressions of anger than those from European and American cultural backgrounds (Adam et al., 2010). This may have led to a greater difference in the present study, which was conducted in Japan, compared with that reported by Derks et al. (2007). Moreover, most participants of the present study were female; they are considered to assess negative emojis more negatively (Jones et al., 2020) and are more sensitive to the appropriateness of the expression (Riordan and Glikson, 2020). This may also lead to more significant variation in the use of emojis for different emotions.

Interestingly, the consistency of emotional valence between emojis and contexts existed only in the context of intense emotional expressions. That is, positive faces were used in positive contexts and negative faces were used in negative contexts only when individuals strongly expressed their emotions. Furthermore, when individuals de-intensified or masked their emotional expressions, the valence of the emoji could differ from that of the context. Thus, Hypothesis H3-2 was only partially supported since the current study did not completely replicate the results of Derks et al. (2007). A surprising finding was that smiling emojis were used when participants de-intensified their expression of negative emotions. This result, however, is consistent with prior studies, which suggested that individuals display more positive expressions during negative disclosures in the presence of others (Friesen, 1972; Lee and Wagner, 2002). Negative expressions are considered “inappropriate” expressions; thus, individuals often suppress or mask them with positive expressions (Lee and Wagner, 2002; Fischer et al., 2003). Particularly, in a cultural context of relatedness and social harmony, the expression of socially disengaging emotions is rather discouraged to foster harmony (Trommsdorff and Heikamp, 2013). Under such cultural restraint on the negative displays, managing expressions through masking is exhibited more regularly in Japan (Friesen, 1972; Li and Shibuya, 2014). Matsumoto and Kudo (1996) also noted that the function of positive expressions, such as smiling, is complicated in Japan as individuals use them to both express happiness and mask inappropriate expressions. Just as a smile displayed when receiving an unsatisfactory gift is an observance of the facial display rules (Goffman, 1967, as cited in Saarni 1984), the smiling emojis used in the negative disclosure chats are more likely to be an observance of similar emoji display rules. In other words, people may downplay the intensity of their expressions when disclosing negative affect and use smiling emojis instead to socially decorate their expressions. Similarly to Kato et al. (2009), the present study found that fewer emojis were used when participants strongly expressed their negative emotions. Additionally, participants used more emojis and fewer emoticons when their positive expressions were intense. This may be because emojis are more emotionally expressive than emoticons (Fischer and Herbert, 2021).

The abovementioned findings suggest that emoji use and emotional expressions are adjusted to targets, social contexts, and expressed emotions similarly to facial displays, which provides insights into the existence of display rules for emojis. This means that some social norms of offline interactions may also be applicable to guide online behaviors. According to Goffman, each situation has a social logic that individuals unconsciously sustain (Goffman, 1959). He stated, “As human beings, we are presumably creatures of variable impulse with moods and energies that change from one moment to the next. As characters put on for an audience, however, we must not be subject to ups and downs.” (Goffman, 1959, p. 63). The present study provides evidence that whether on the “stage” of FTF or CMC, individuals present themselves according to the situation or impression they wish to project. Thus, as an “expressive tool” with a high degree of social information (Erle et al., 2021), emojis may be more social than previously thought. Moreover, while the degree of emotional expression was not considered in prior studies, it is an essential factor in emoji use that cannot be regarded as a substitute for frequency of use.

The final hypothesis postulated that emotional expressions using emojis would be positively associated with well-being, whereas emotion management with emojis would demonstrate a weak negative association with well-being. Regression analysis revealed that individuals who expressed emotions with emojis were more likely to report more subjective well-being and fewer depressive symptoms. Conversely, individuals who managed their expressions according to emotion rules were more likely to report depressive symptoms. However, emotion management with emojis was a relatively weak predictor of depressive symptoms, with marginal statistical significance (*p* = 0.07). It is argued that results with *p*-values between 0.05 and 0.1 are not very evidential (Olsson-Collentine et al., 2019) but can trigger future studies on this potential correlation. A possible cultural factor should also be considered, wherein managing emotions to maintain interpersonal harmony is common and encouraged in Japan, which may result in less deleterious outcomes, as predicted (Matsumoto et al., 2008a; Schouten et al., 2020). Furthermore, the R^2^ values of regressions were extremely low. However, the regression models were significant, and a low R^2^ value in social science research does not necessarily mean that the effect can be ignored and is unimportant (Moksony, 1999). For example, despite the low value R^2^ values, the present study has effectively replicated previous results underscoring that emotional expression is associated with a healthy mind (Gable et al., 2004), as well as Vuillier et al.,'s (2018) findings, which demonstrate that using more emojis increases happiness online. Thus, although H4 is generally supported, the current findings should be treated with caution, and it is important for future research to explore the psychological impact of emotion management in digital contexts, for example, examining possible mediators or distinguishing between surface and deep acting, to determine the reproducibility of the present results.

As reported in the data analysis, positive correlations were found between age, emoji frequency, and emotional expression, whereas a negative correlation was observed between age and emotion management. The correlations between age with emoji usage and emotional expression could have emerged as a result of the increased opportunity to socially interact and express feelings when one entered college or began working. On the other hand, even though display rule endorsement generally increased with age among Japanese individuals (Wice et al., 2019), Zimmermann and Iwanski (2014) reported a decline in emotion regulation strategies for certain emotions during mid-adolescence and emerging adulthood compared with early adolescence. A possible explanation is that, because of the increase in social support seeking during emerging adulthood and reorganization of regulation strategies during middle adolescence, a negative correlation between age and emotion management may arise at certain periods (Zimmermann and Iwanski, 2014).

Emojis are important tools for expressing emotions in cyberspace. In response to the increase in emotional expressions in cyberspace, more focus is required on the impact of emotion management using emojis on mental health. Thus, future research could investigate whether other cultures show similar results to verify whether this weak association is related to cultural differences in the display rules. Moreover, the results underscore that different emoji usage or mindsets can lead to various psychological effects. Particularly, the demand to “act” according to social rules might be detrimental for some adolescents.

### 5.1. Limitations and future directions

The most notable limitation of this study is the gender imbalance; almost all participants were female, which limits the generalizability of the findings and does not allow inferences about sex difference. This may be because most users of “Simeji,” the keyboard app released for the survey, are female (MarkeZine, 2016), as well as because of the possibility that women are more willing to volunteer (Dickinson et al., 2012). Indeed, women use more emotion regulation strategies (Greenaway et al., 2018) and are more sensitive to the appropriateness of emotional expressions (Riordan and Glikson, 2020). Furthermore, women are more likely to feel upset about violated politeness (Herring, 2000). These gender differences may have led to the observed differences in emotional expressions being more significant than they actually are. Thus, future research is required to investigate the gender differences in emoji display rules and examine whether similar rules can be observed in the gender-balanced samples. However, gender-imbalanced samples are sometimes more representative of groups with a quantitative gender disbalance (Dickinson et al., 2012). For example, the present study reflects to some extent the characteristics of “Simeji,” which is dominated by female users (MarkeZine, 2016), and the fact that women use emojis more frequently (Kato, 2017; Butterworth et al., 2019). Additionally, the results of the current study may also be limited to younger populations. Emotion management strategies vary across ages (Zimmermann and Iwanski, 2014; Wice et al., 2019). Younger individuals are also more familiar with emojis (Cui, 2022) and are better at adapting emoji use to different settings. Therefore, future studies ought to be conducted with other age groups and compare the age differences. Overall, it should be noted that the current findings were gained through a survey of “Simeji” users, and while the results may contribute to the understanding of populations with similar characteristics, further investigations with different samples are warranted to evaluate the reproducibility.

Regarding the research method, the present results relied on self-reports from participants. This also suggests that emoji rules may be applied at a more conscious level. Because of the high accessibility, self-reported measures are frequently used in prior research to investigate emotional expressivity (e.g., Zhao, 2002; Pollastri et al., 2018). However, while the subjective feelings may change according to contexts (Hochschild, 1983), whether individuals automatically experience different emotions or subconsciously adjust their use of emojis based on the context remains unclear. Thus, future research could explore emotional behaviors or experiences that are uncontrollable for participants when they use emojis. Second, this study controlled for verbal information in online chats. However, analyzing verbal and nonverbal behavior separately is of limited value (Lee and Wagner, 2002). Indeed, individuals choose emojis in their daily chats according to the text messages. Walther and D’Addario (2001) also indicated that emojis are overwhelmed by sentiments in the accompanying texts. Thus, allowing individuals to enter both messages and emojis would be more closely related to their actual use. Third, because participants used different devices and the display of emojis varied in different operating systems, the emoji categories of the Unicode standard may not completely reflect the emotional valence of the emojis. Research on the universal and standardized emotional valences of emojis is thus needed to further explore emotional expression using emojis. Finally, to reduce the burden on participants, only parts of the CES-D were used when measuring depressive symptoms; subjective well-being was also assessed with one item, which did not allow checking for any reliability. These may have had some impact on the results, calling for future research to use a more complete mental health scale to analyze the relationship between emotion management using emojis and well-being.

In terms of the research content, only positive and negative emotions were included. However, prior research suggests that different positive or negative emotions correspond to different display rules (Matsumoto et al., 2005, 2008a). For example, individuals often hide fear and disgust while they neutralize or de-amplify sadness and anger (Diefendorff and Greguras, 2009). Therefore, future studies could subdivide emotions and examine other variations of expressing different emotions. Besides, the results of this study may be related to the Japanese cultural context. Cultural differences regarding context differentiation and display rules have been early suggested (Ekman, 1970; Friesen, 1972; Matsumoto et al., 2009). Recent Studies also showed that different cultures might have different paradigms for emoji usage (Guntuku et al., 2019). Thus, future research should investigate the effect of cultural differences: that is, whether other cultures exhibit identical results; how the association between emotion management and depressive symptoms is related to cultural differences of the display rules; and how individuals in other cultures use different categories of emojis dependent on the recipients.

## 6. Conclusion

In summary, this study takes the first step in examining how emotional expressions and specific emoji categories are adjusted to different contexts and how emotion management is related to mental health. The emotion rules for emojis and facial displays are similar; they guide individuals to follow social situations to express themselves appropriately. Individuals adapt their expressions for different targets, contextual values, and public or private settings. The emotional valence of emojis is consistent with contexts only when emotions are strongly expressed. When individuals attenuate emotional expressions, they use euphemistic emojis and those with different emotional values depending on contexts. Interestingly, smiling faces do not necessarily express happiness but are also used to de-intensify and mask emotions that are not appropriate for expression. The results also revealed that expressing emotions with emojis is associated with subjective well-being. In contrast, managing emotions with emojis is weakly associated with depressive symptoms. The present findings indicate that the degree of emotional expression is an essential element for emoji research and provides directions for exploring more emotion norms from this perspective. Finally, the present study emphasizes that the psychological impact of online emotion rules cannot be ignored and warrants further research.

## Data availability statement

The raw data supporting the conclusions of this article will be made available by the author, without undue reservation.

## Ethics statement

The studies involving human participants were reviewed and approved by the III/GSII, the University of Tokyo. Written informed consent to participate in this study was provided by the participants or their legal guardian/next of kin.

## Author contributions

The author confirms being the sole contributor of this work and has approved it for publication.

## Conflict of interest

The author declares that the research was conducted in the absence of any commercial or financial relationships that could be construed as a potential conflict of interest.

## Publisher’s note

All claims expressed in this article are solely those of the author and do not necessarily represent those of the author’s affiliated organizations, or those of the publisher, the editors and the reviewers. Any product that may be evaluated in this article, or claim that may be made by its manufacturer, is not guaranteed or endorsed by the publisher.

## References

[ref1] Adam H. Shirako A. Maddux W. W. (2010). Cultural variance in the interpersonal effects of anger in negotiations. Psychol. Sci. 21, 882–889. doi: 10.1177/0956797610370755, PMID: 20483822

[ref2] Agresti A. (2002). Categorical data analysis (2nd Ed.). New York: Wiley, doi: 10.1002/0471249688

[ref3] Al Rashdi F. (2018). Functions of emojis in Whats app interaction among Omanis. Disc. Cont. Media 26, 117–126. doi: 10.1016/j.dcm.2018.07.001

[ref4] Alt M. (2016). The secret lives of Emoji: How emoticons conquered the world. Kindle Edn Amazon Digital Services.

[ref5] Arakawa A. Suzuki N. (2004). Effects of emoticon with an apologetic message on the receiver’s feelings (in Japanese). Interp. Soc. Psychol. Res. 4, 135–140. doi: 10.18910/3801

[ref6] Bai Q. Dan Q. Mu Z. Yang M. (2019). A systematic review of emoji: current research and future perspectives. Front. Psychol. 10:222. doi: 10.3389/fpsyg.2019.02221 31681068 PMC6803511

[ref7] Boutet I. LeBlanc M. Chamberland J. A. Collin C. A. (2021). Emojis influence emotional communication, social attributions, and information processing. Comput. Hum. Behav. 119:106722. doi: 10.1016/j.chb.2021.106722

[ref8] Butterworth S. E. Giuliano T. A. White J. Cantu L. Fraser K. C. (2019). Sender gender influences Emoji interpretation in text messages. Front. Psychol. 10:784. doi: 10.3389/fpsyg.2019.00784 31024407 PMC6459937

[ref9] Cahyaningtyas R. M. Kusumaningrum R. Sutikno S. Riyanto D. E. (2017). “Emotion detection of tweets in Indonesian language using LDA and expression symbol conversion” in Paper presented at the 2017 1st international conference on informatics and computational sciences (ICICoS): (IEEE Semarang). doi: 10.1109/ICICOS.2017.8276371

[ref10] Callender J. Bridge P. Al-Samarraie F. Blair D. (2022). The use of emoji to establish student wellbeing: does the image reflect the reality? J. Radiother. Pract. 22:704. doi: 10.1017/S1460396921000704

[ref11] Calvo R. A. Peters D. (2014). Positive computing: Technology for Wellbeing and Human Potential. Cambridge, Massachusetts: MIT Press, doi: 10.7551/mitpress/9764.001.0001

[ref12] Carminati L. (2021). Emotions, emotion management and emotional intelligence in the workplace: healthcare professionals' experience in emotionally-charged situations. Front. Sociol. 6:640384. doi: 10.3389/fsoc.2021.640384 33889607 PMC8055814

[ref13] Cheshin A. (2020). The impact of non-normative displays of emotion in the workplace: how inappropriateness shapes the interpersonal outcomes of emotional displays. Front. Psychol. 11:6. doi: 10.3389/fpsyg.2020.00006, PMID: 32116884 PMC7033655

[ref14] Cheung R. Y. Park I. J. (2010). Anger suppression, interdependent self-construal, and depression among Asian American and European American college students. Cultur. Divers. Ethnic Minor. Psychol. 16, 517–525. doi: 10.1037/a0020655, PMID: 21058815 PMC3058745

[ref15] Chun H. R. Cho I. Choi Y. Cho S. I. (2020). Effects of emotional labor factors and working environment on the risk of depression in pink-collar workers. Int. J. Environ. Res. Public Health 17:5208. doi: 10.3390/ijerph17145208 32707657 PMC7400525

[ref16] Collins N. L. Miller L. C. (1994). Self-disclosure and liking: a meta-analytic review. Psychol. Bull. 116, 457–475. doi: 10.1037/0033-2909.116.3.457, PMID: 7809308

[ref17] Coyle M. A. Carmichael C. L. (2019). Perceived responsiveness in text messaging: the role of emoji use. Comput. Hum. Behav. 99, 181–189. doi: 10.1016/j.chb.2019.05.023

[ref18] Cui J. (2022). Respecting the old and loving the young: emoji-based sarcasm interpretation between younger and older adults. Front. Psychol. 13:897153. doi: 10.3389/fpsyg.2022.897153, PMID: 35664181 PMC9161290

[ref19] Derks D. Bos A. E. R. von Grumbkow J. (2007). Emoticons and social interaction on the internet: the importance of social context. Comput. Hum. Behav. 23, 842–849. doi: 10.1016/j.chb.2004.11.013

[ref20] Derks D. Fischer A. H. Bos A. E. R. (2008). The role of emotion in computer-mediated communication: a review. Comput. Hum. Behav. 24, 766–785. doi: 10.1016/j.chb.2007.04.004

[ref21] Dickinson E. R. Adelson J. L. Owen J. (2012). Gender balance, representativeness, and statistical power in sexuality research using undergraduate student samples. Arch. Sex. Behav. 41, 325–327. doi: 10.1007/s10508-011-9887-1, PMID: 22228196

[ref22] Diefendorff J. M. Greguras G. J. (2009). Contextualizing emotional display rules: examining the roles of targets and discrete emotions in shaping display rule perceptions. J. Manag. 35, 880–898. doi: 10.1177/0149206308321548

[ref23] Dzokoto V. A. Osei-Tutu A. Kyei J. J. Twum-Asante M. Attah D. A. Ahorsu D. K. (2018). Emotion norms, display rules, and regulation in the Akan society of Ghana: an exploration using proverbs. Front. Psychol. 9:1916. doi: 10.3389/fpsyg.2018.01916 30429804 PMC6220724

[ref24] Ekman P. (1970). Universal facial expressions of emotion. Calif. Ment. Health Res. Digest. 8, 151–158.

[ref25] Ekman P. (1972). “Universal and cultural differences in facial expression of emotions” in Nebraska symposium on motivation. ed. Cole J. (Lincoln: University of Nebraska Press), 207–283.

[ref26] Ekman P. Friesen W. V. (1969). The repertoire of nonverbal behavior: categories, origins, usage, and coding. Semiotica 1, 49–98. doi: 10.1515/semi.1969.1.1.49

[ref27] Ekman P. Friesen W. V. (1975). Unmasking the face: A guide to recognizing emotions from facial clues. Englewood Cliffs, NJ: Prentice-Hall.

[ref28] Ekman P. Friesen W. V. (1978). Facial action coding system: A technique for the measurement of facial movement. California, CA: Consulting Psychologists Press, Palo Alto.

[ref29] Emogi. (2015). Emoji report. Available at: https://cdn.emogi.com/docs/reports/2015_emoji_report.pdf

[ref30] Erle T. M. Schmid K. Goslar S. H. Martin J. D. (2021). Emojis as social information in digital communication. Emotion 22, 1529–1543. doi: 10.1037/emo0000992 34351198

[ref31] Evers C. Fischer A. H. Mosquera P. M. Manstead A. S. R. (2005). Anger and social appraisal: a “spicy” sex difference? Emotion 5, 258–266. doi: 10.1037/1528-3542.5.3.258 16187862

[ref32] Felbo B. Mislove A. Søgaard A. Rahwan I. Lehmann S. (2017). Using millions of emoji occurrences to learn any-domain representations for detecting sentiment, emotion, and sarcasm. Paper presented at the 2017 conference on empirical methods in natural language processing. Copenhagen: Association for Computational Linguistics. doi: 10.18653/v1/D17-1169

[ref33] Fischer B. Herbert C. (2021). Emoji as affective symbols: affective judgments of emoji, emoticons, and human faces varying in emotional content. Front. Psychol. 12:645173. doi: 10.3389/fpsyg.2021.645173 33959074 PMC8093811

[ref34] Fischer A. H. Manstead A. S. R. Zaalberg R. (2003). Social influences on the emotion process. Eur. Rev. Soc. Psychol. 14, 171–201. doi: 10.1080/10463280340000054

[ref35] Fridlund A. J. (1991). Sociality of solitary smiling: potentiation by an implicit audience. J. Pers. Soc. Psychol. 60, 229–240. doi: 10.1037/0022-3514.60.2.229

[ref36] Friedman H. S. Miller-Herringer T. (1991). Nonverbal display of emotion in public and in private: self-monitoring, personality, and expressive cues. J. Pers. Soc. Psychol. 61, 766–775. doi: 10.1037/0022-3514.61.5.766 1753331

[ref37] Friesen W. V. (1972). Cultural differences in facial expressions in a social situation: An experimental test of the concept of display rules. University of California, San Francisco.

[ref38] Fussell S. (2002). The verbal communication of emotion: Interdisciplinary perspectives. Mahwah, NJ: Lawrence Erlbaum Associates Publishers. doi: 10.4324/9781410606341

[ref39] Gable S. L. Reis H. T. Impett E. A. Asher E. R. (2004). What do you do when things go right? The intrapersonal and interpersonal benefits of sharing positive events. J. Pers. Soc. Psychol. 87, 228–245. doi: 10.1037/0022-3514.87.2.228 15301629

[ref40] Gabriel A. S. Cheshin A. Moran C. M. van Kleef G. A. (2016). Enhancing emotional performance and customer service through human resources practices: a systems perspective. Hum. Resour. Manag. Rev. 26, 14–24. doi: 10.1016/j.hrmr.2015.09.003

[ref41] Glikson E. Cheshin A. van Kleef G. A. (2018). The dark side of a smiley: effects of smiling emoticons on virtual first impressions. Soc. Psychol. Personal. Sci. 9, 614–625. doi: 10.1177/1948550617720269

[ref42] Goffman E. (1959). The presentation of self in everyday life. London: Penguin Books.

[ref43] Grandey A. A. (2003). When “the show must go on”: surface acting and deep acting as determinants of emotional exhaustion and peer-rated service delivery. Acad. Manag. J. 46, 86–96. doi: 10.2307/30040678

[ref44] Grattet R. (2011). Societal reactions to deviance. Annu. Rev. Sociol. 37, 185–204. doi: 10.1146/annurev-soc-081309-150012

[ref45] Greenaway K. H. Kalokerinos E. K. Williams L. A. (2018). Context is everything (in emotion research). Soc. Personal. Psychol. Compass 12:e12393. doi: 10.1111/spc3.12393

[ref46] Guntuku S. C. Li M. Tay L. Ungar L. H. (2019). Studying cultural differences in emoji usage across the east and the west. Proc. Int. AAAI Conf. Web Soc. Media 13, 226–235. doi: 10.1609/icwsm.v13i01.3224

[ref47] Hancock J. T. Landrigan C. Silver C. (2007). Expressing emotion in text-based communication. Proc. SIGCHI Conf. Hum. Fact. Comp. Syst. 929:764. doi: 10.1145/1240624.1240764

[ref48] Harada T. (2004). The role of “face marks” in promoting smooth communication and expressing consideration and politeness in Japanese (in Japanese). Lang. Cult. 8, 205–224. doi: 10.14990/00000403

[ref49] Herring S. C. (2000). Gender differences in CMC: findings and implications. Comput. Prof. Soc. Respons. J. 18 http://archive.cpsr.net/publications/newsletters/issues/2000/winter2000/herring.html

[ref50] Hiroya Y. (2007). The effects of emotional expressiveness on ratings of behavior and a prediction of future relations in close interpersonal situations (in Japanese). Bull. Inst. Hum. Sci. Toyo Univ. 7, 297–307.

[ref51] Hochschild A. (1983). The managed heart: The commercialization of human feeling. Berkeley, CA: University of California Press.

[ref52] Honna N. Hoffer B. (1986). An English dictionary of Japanese culture. Tokyo: Yuhikaku.

[ref53] Hülsheger U. R. Lang J. W. Maier G. W. (2010). Emotional labor, strain, and performance: testing reciprocal relationships in a longitudinal panel study. J. Occup. Health Psychol. 15, 505–521. doi: 10.1037/a0021003, PMID: 21058862

[ref54] IBM. (2020). Interpreting adjusted residuals in crosstabs cell statistics. Available at: https://www.ibm.com/support/pages/interpreting-adjusted-residuals-crosstabs-cell-statistics.

[ref55] Inoue M. (2008). Emotional features based on experiences of the falsifying of emotions (in Japanese). Jpn. J. Res. Emot. 15, 71–79. doi: 10.4092/jsre.15.71

[ref56] Ip K. I. Miller A. L. Karasawa M. Hirabayashi H. Kazama M. Wang L.. (2021). Emotion expression and regulation in three cultures: Chinese, Japanese, and American preschoolers’ reactions to disappointment. J. Exp. Child Psychol. 201:104972. doi: 10.1016/j.jecp.2020.104972 32919326 PMC7583664

[ref57] Jakobs E. B. Manstead A. S. R. Fischer A. H. (2001). Social context effects on facial activity in a negative emotional setting. Emotion 1, 51–69. doi: 10.1037/1528-3542.1.1.51, PMID: 12894811

[ref58] Japanese Cabinet Office. (2012). Results of the first quality of life survey (in Japanese). Available at: https://www5.cao.go.jp/keizai2/koufukudo/shiryou/7shiryou/3.pdf.

[ref59] Jones L. L. Wurm L. H. Norville G. A. Mullins K. L. (2020). Sex differences in emoji use, familiarity, and valence. Comput. Hum. Behav. 108:106305. doi: 10.1016/j.chb.2020.106305

[ref60] Judge T. A. Woolf E. F. Hurst C. (2009). Is emotional labor more difficult for some than for others? A multilevel, experience-sampling study. Pers. Psychol. 62, 57–88. doi: 10.1111/j.1744-6570.2008.01129.x

[ref61] Kato Y. (2017). Basic survey on the use of LINE stamps (in Japanese). J. Inf. Media Stud. 3, 21–34. https://cir.nii.ac.jp/crid/1050282812823049088

[ref62] Kato Y. Kato S. Akahori K. (2005). Analysis of emotional aspects in e−mail communication by mobile phone with a teacher or a friend (in Japanese). Jpn. Soc. Educ. Inform. 21, 543–557. doi: 10.20694/jjsei.21.3_3

[ref63] Kato S. Kato Y. Scott D. (2009). Relationships between emotional states and emoticons in mobile phone email communication in Japan (in Japanese). Int. J. E Learn. 8, 385–401.

[ref64] Kaye L. K. Wall H. J. Malone S. A. (2016). ‘Turn that frown upside-down’: a contextual account of emoticon usage on different virtual platforms. Comput. Hum. Behav. 60, 463–467. doi: 10.1016/j.chb.2016.02.088

[ref65] Kim H. J. Choo J. (2017). Emotional labor: links to depression and work-related musculoskeletal disorders in call center workers. Workplace. Health. Saf. 65, 346–354. doi: 10.1177/2165079916667512 27895237

[ref66] Kito M. (2005). Self-disclosure in romantic relationships and friendships among American and Japanese college students. J. Soc. Psychol. 145, 127–140. doi: 10.3200/SOCP.145.2.127-140 15816343

[ref67] Krekhov A. Emmerich K. Fuchs J. Krueger J. H. (2022). Interpolating happiness: understanding the intensity gradations of face emojis across cultures. CHI Conference on Human Factors in Computing Systems 1:7661. doi: 10.1145/3491102.3517661

[ref68] Larsen R. J. Diener E. (1987). Affect intensity as an individual difference characteristic: a review. J. Res. Pers. 21, 1–39. doi: 10.1016/0092-6566(87)90023-7

[ref69] Lee Y. J. Matsumoto Y. (2011). Emotional display rules of Japanese and Koreans (in Japanese). Shinrigaku Kenkyu 82, 415–423. doi: 10.4992/jjpsy.82.415 22319949

[ref70] Lee M. Pekrun R. Taxer J. L. Schutz P. A. Vogl E. Xie X. (2016). Teachers’ emotions and emotion management: integrating emotion regulation theory with emotional labor research. Soc. Psychol. Educ. 19, 843–863. doi: 10.1007/s11218-016-9359-5

[ref71] Lee V. Wagner H. (2002). The effect of social presence on the facial and verbal expression of emotion and the interrelationships among emotion components. J. Nonverbal Behav. 26, 3–25. doi: 10.1023/A:1014479919684

[ref72] Li S. Shibuya S. (2014). Consciousness on expressions of social laughter in Japanese and Chinese undergraduate and graduate students (in Japanese). Mejiro J. Psychol. 10, 25–38.

[ref73] Liu S. Sun R. (2020). To express or to end? Personality traits are associated with the reasons and patterns for using emojis and stickers. Front. Psychol. 11:1076. doi: 10.3389/fpsyg.2020.01076 32581941 PMC7296135

[ref74] Lo S. K. (2008). The nonverbal communication functions of emoticons in computer-mediated communication. Cyberpsychol. Behav. 11, 595–597. doi: 10.1089/cpb.2007.0132 18817486

[ref75] Lu X. Ai W. Chen Z. Cao Y. Mei Q. (2022). Emojis predict dropouts of remote workers: an empirical study of emoji usage on GitHub. PLoS One 17:e0261262. doi: 10.1371/journal.pone.0261262 35081111 PMC8791473

[ref76] Lu X. Ai W. Liu X. Li Q. Wang N. Huang G.. (2016). Learning from the ubiquitous language: An empirical analysis of emoji usage of smartphone users. Proceedings of the 2016 ACM international joint conference on pervasive and ubiquitous computing Association for Computing Machinery’, 770–780. doi: 10.1145/2971648.2971724

[ref77] Luor T. T. Wu L. L. Lu H. P. Tao Y. H. (2010). The effect of emoticons in simplex and complex task-oriented communication: an empirical study of instant messaging. Comput. Hum. Behav. 26, 889–895. doi: 10.1016/j.chb.2010.02.003

[ref78] Manago A. M. McKenzie J. (2022). “Culture and digital media in adolescent development” in Handbook of adolescent digital media use and mental health. eds. Nesi J. Telzer E. H. Prinstein M. J. (Cambridge: Cambridge University Press), 162–187. doi: 10.1017/9781108976237.010

[ref79] MarkeZine. (2016). Popular application ‘Simeji’ among young generation, 3 essential measures of the marketing (in Japanese). Available at: https://markezine.jp/article/detail/24763

[ref80] Markus H. R. Kitayama S. (1991). Culture and the self: implications for cognition, emotion, and motivation. Psychol. Rev. 98, 224–253. doi: 10.1037/0033-295X.98.2.224

[ref81] Matsumoto D. (1990). Cultural similarities and differences in display rules. Motiv. Emot. 14, 195–214. doi: 10.1007/BF00995569

[ref82] Matsumoto D. Kudo C. (1996). The emotion world of the Japanese-solving mysterious cultural enigmas (in Japanese). Tokyo: SeishinShobo.

[ref83] Matsumoto D. Takeuchi S. Andayani S. Kouznetsova N. Krupp D. (1998). The contribution of individualism–collectivism to cross-national differences in display rules. Asian J. Soc. Psychol. 1, 147–165. doi: 10.1111/1467-839X.00010

[ref84] Matsumoto D. Yoo S. H. Fontaine J. (2009). Hypocrisy or maturity? Culture and context differentiation. Eur. J. Personal. 23, 251–264. doi: 10.1002/per.716

[ref87] Matsumoto D. Yoo S. H. Fontaine J. Anguas-Wong A. M. Arriola M. Ataca B. (2008a). Mapping expressive differences around the world: the relationship between emotional display rules and individualism versus collectivism. J. Cross-Cult. Psychol. 39, 55–74. doi: 10.1177/0022022107311854

[ref86] Matsumoto D. Yoo S. H. Hirayama S. Petrova G. (2005). Development and validation of a measure of display rule knowledge: the display rule assessment inventory. Emotion 5, 23–40. doi: 10.1037/1528-3542.5.1.23 15755217

[ref85] Matsumoto D. Yoo S. H. Nakagawa S. 37 members of the Multinational Study of Cultural Display Rules (2008b). Culture, emotion regulation, and adjustment. J. Pers. Soc. Psychol. 94, 925–937. doi: 10.1037/0022-3514.94.6.925 18505309

[ref88] McLaughlin C. Vitak J. (2012). Norm evolution and violation on Facebook. New Media Soc. 14, 299–315. doi: 10.1177/1461444811412712

[ref89] Mesch G. S. Beker G. (2010). Are norms of disclosure of online and offline personal information associated with the disclosure of personal information online? Hum. Commun. Res. 36, 570–592. doi: 10.1111/j.1468-2958.2010.01389.x

[ref90] Mesmer-Magnus J. R. Dechurch L. A. Wax A. (2012). Moving emotional labor beyond surface and deep acting a discordance–congruence perspective. Organ. Psychol. Rev. 2, 6–53. doi: 10.1177/2041386611417746

[ref91] Ministry of Internal Affairs and Communications of Japan. (2021). White paper on information and communications in Japan. Available at: https://www.soumu.go.jp/johotsusintokei/whitepaper/eng/WP2021/2021-index.html

[ref92] Moksony F. (1999). Small is beautiful. The use and interpretation of R^2^ in social research. Szociológiai Szemle, 130–138.

[ref93] Moran C. M. Diefendorff J. M. Greguras G. J. (2013). Understanding emotional display rules at work and outside of work: the effects of country and gender. Motiv. Emot. 37, 323–334. doi: 10.1007/s11031-012-9301-x

[ref94] Morry M. M. (2005). Allocentrism and friendship satisfaction: the mediating roles of disclosure and closeness. Can. J. Behav. Sci. 37, 211–222. doi: 10.1037/h0087258

[ref95] Nakamura M. (1991). Display and decoding rules in the communication of emotion: a conceptual analysis and a cross-cultural questionnaire study (in Japanese). Bull. Fac. Hum. Sci. Osaka Univ.. 17, 115–145. doi: 10.18910/7911

[ref96] Nomura S. Endo K. Omori T. Kisugi N. (2022). Changes in parental involvement and perceptions in parents of young children during the COVID-19 pandemic: a cross-sectional observational study in Japan. Glob. Health. Med. 4, 166–173. doi: 10.35772/ghm.2022.01003 35855065 PMC9243405

[ref97] Olsson-Collentine A. Van Assen M. A. Hartgerink C. H. (2019). The prevalence of marginally significant results in psychology over time. Psychol. Sci. 30, 576–586. doi: 10.1177/0956797619830326, PMID: 30789796 PMC6472145

[ref98] Pennebaker J. W. Zech E. Rimé B. (2001). “Disclosing and sharing emotion: psychological, social, and health consequences” in Handbook of bereavement research: Consequences, coping, and care. eds. Stroebe M. S. Hansson R. O. Stroebe W. Schut H. (Washington, DC: American Psychological Association), 517–543. doi: 10.1037/10436-022

[ref99] Pollastri A. R. Raftery-Helmer J. N. Cardemil E. V. Addis M. E. (2018). Social context, emotional expressivity, and social adjustment in adolescent males. Psychol. Men Masculinity 19, 69–77. doi: 10.1037/men0000081

[ref100] Rice R. E. Love G. (1987). Electronic emotion: socioemotional content in a computer-mediated communication network. Commun. Res. 14, 85–108. doi: 10.1177/009365087014001005

[ref101] Riordan M. A. (2017). Emojis as tools for emotion work: communicating affect in text messages. J. Lang. Soc. Psychol. 36, 549–567. doi: 10.1177/0261927X17704238

[ref102] Riordan M. A. Glikson E. (2020). On the hazards of the technology age: How using emojis affects perceptions of leaders. Int. J. Bus. Commun, 1–22. doi: 10.1177/2329488420971690

[ref103] Ruel E. Wagner W. Gillespie B. (2016). “Pretesting and pilot testing” in The practice of survey research: Theory and applications (California: SAGE Publications, Inc.), 101–119. doi: 10.4135/9781483391700

[ref104] Saarni C. (1984). An observational study of children’s attempts to monitor their expressive behavior. Child Dev. 55, 1504–1513. doi: 10.2307/1130020

[ref105] Safdar S. Friedlmeier W. Matsumoto D. Yoo S. H. Kwantes C. T. Kakai H.. (2009). Variations of emotional display rules within and across cultures: a comparison between Canada, United States, and Japan. Can. J. Behav. Sci. Rev. Canadienne Sci. Comport. 41, 1–10. doi: 10.1037/a0014387

[ref106] Schouten A. Boiger M. Kirchner-Häusler A. Uchida Y. Mesquita B. (2020). Cultural differences in emotion suppression in Belgian and Japanese couples: a social functional model. Front. Psychol. 11:1048. doi: 10.3389/fpsyg.2020.01048 32670134 PMC7326130

[ref107] Sharpe D. (2015). Chi-square test is statistically significant: now what? Pract. Assess. Res. Eval. 20:148. doi: 10.7275/tbfa-x148

[ref108] Shi H. Liu X. Li K. Xie J. (2019). “Emoji usage and interpersonal relationship in computer-mediated communication” in International joint conference on information, media and engineering (IJCIME) (IEEE Publications), 262–266. doi: 10.1109/IJCIME49369.2019.00059

[ref109] Shields S. A. (2005). The politics of emotion in everyday life: “appropriate” emotion and claims on identity. Rev. Gen. Psychol. 9, 3–15. doi: 10.1037/1089-2680.9.1.3

[ref110] Shoeb A. de Melo G. (2020). Are emojis emotional? A study to understand the association between emojis and emotions. Available at: https://arxiv.org/abs/2005.00693v1.

[ref111] Sloan M. M. (2012). Controlling anger and happiness at work: an examination of gender differences. Gend. Work. Organ. 19, 370–391. doi: 10.1111/j.1468-0432.2010.00518.x

[ref112] Sproull L. Kiesler S. (1986). Reducing social context cues: electronic mail in organizational communication. Manag. Sci. 32, 1492–1512. doi: 10.1287/mnsc.32.11.1492

[ref113] Sugiura T. Umaoka K. (2003). Cognitive control and depression in female university students. Jpn. J. Health Psychol. 16, 31–42. doi: 10.11560/jahp.16.1_31

[ref114] Takehara T. Sato N. (2004). Promotion effect of emotional communication by joyful emoticons (in Japanese). Japan. Acad. Fac. Stud. 4, 9–17.

[ref115] Teh P. L. Boon O. P. Gill C. M. (2020). “Relationships between emoticon usage and recipient groups in students’ text messages” in Proceedings of the 22nd International Conference on Information Integration and Web-Based Applications & Services, 300–304. doi: 10.1145/3428757.3429127

[ref116] Timmers M. Fischer A. H. Manstead A. S. R. (1998). Gender differences in motives for regulating emotions. Personal. Soc. Psychol. Bull. 24, 974–985. doi: 10.1177/0146167298249005

[ref117] Trommsdorff G. Heikamp T. (2013). “Socialization of emotions and emotion regulation in cultural context” in Cultural variations in psychopathology: From research to practice. eds. Barnow S. Balkir N. (Boston: Hogrefe Publishing), 67–92.

[ref118] Uchida Y. Townsend S. S. Rose Markus H. R. Bergsieker H. B. (2009). Emotions as within or between people? Cultural variation in lay theories of emotion expression and inference. Personal. Soc. Psychol. Bull. 35, 1427–1439. doi: 10.1177/0146167209347322 19745200

[ref119] Van Dam L. Rietstra S. Van der Drift E. Stams G. J. J. M. Van der Mei R. Mahfoud M.. (2019). Can an emoji a day keep the doctor away? An explorative mixed-methods feasibility study to develop a self-help app for youth with mental health problems. Front. Psych. 10:593. doi: 10.3389/fpsyt.2019.00593 PMC6716472 31507464

[ref120] Vidal L. Ares G. Jaeger S. R. (2016). Use of emoticon and emoji in tweets for food-related emotional expression. Food Qual. Prefer. 49, 119–128. doi: 10.1016/j.foodqual.2015.12.002

[ref121] Vuillier L. Brooks A. W. Norton M. I. (2018). Amount and diversity of digital emotional expression predicts happiness. Boston, MA: Harvard Business School.

[ref122] Wagner H. L. Lee V. (1999). “Facial behavior alone and in the presence of others” in The social context of nonverbal behavior. eds. Philippot P. Feldman R. S. Coats E. J. (Cambridge: Cambridge University Press), 262–286.

[ref123] Wagner H. L. Smith J. (1991). Facial expression in the presence of friends and strangers. J. Nonverbal Behav. 15, 201–214. doi: 10.1007/BF00986922

[ref124] Walther J. B. D’Addario K. P. (2001). The impacts of emoticons on message interpretation in computer-mediated communication. Soc. Sci. Comput. Rev. 19, 324–347. doi: 10.1177/089443930101900307

[ref125] Weisberg Y. J. DeYoung C. G. Hirsh J. B. (2011). Gender differences in personality across the ten aspects of the big five. Front. Psychol. 2:178. doi: 10.3389/fpsyg.2011.00178 21866227 PMC3149680

[ref126] Wice M. Matsui T. Tsudaka G. Karasawa M. Miller J. G. (2019). Verbal display rule knowledge: a cultural and developmental perspective. Cogn. Dev. 52:100801. doi: 10.1016/j.cogdev.2019.100801

[ref127] Wolf A. (2000). Emotional expression online: gender differences in emoticon use. Cyber Psychol. Behav. 3, 827–833. doi: 10.1089/10949310050191809

[ref128] Xu L. Yi C. Xu Y. (2007). Emotional expression online: the impact of task, relationship and personality perception on emoticon usage in instant messenger. PACIS Proc. 79 https://aisel.aisnet.org/pacis2007/79/

[ref129] Yamamoto K. Kimura M. (2017). The influence of interrelationship on emoticon use in LINE (in Japanese). Jpn. J. Res. Emot. 25:ps30. doi: 10.4092/jsre.25.Supplement_ps30

[ref130] Yigit O. T. (2005). Emoticon usage in task-oriented and socioemotional contexts in online-discussion boards. Tallahassee (FL): Florida State University.

[ref131] Zapf D. (2002). Emotion work and psychological well-being. Hum. Resour. Manag. Rev. 12, 237–268. doi: 10.1016/S1053-4822(02)00048-7

[ref132] Zhang H. Zhou Z. E. Zhan Y. Liu C. Zhang L. (2018). Surface acting, emotional exhaustion, and employee sabotage to customers: moderating roles of quality of social exchanges. Front. Psychol. 9:2197. doi: 10.3389/fpsyg.2018.02197 30487768 PMC6246630

[ref133] Zhao S. (2002). A comparative study on display rules between Chinese and Japanese (in Japanese). J. Intercult. Stud. 6, 77–89.

[ref134] Zimmermann P. Iwanski A. (2014). Emotion regulation from early adolescence to emerging adulthood and middle adulthood: age differences, gender differences, and emotion-specific developmental variations. Int. J. Behav. Dev. 38, 182–194. doi: 10.1177/0165025413515405

