# Supplementary material for: Are you really smiling? Display rules for emojis and the relationship between emotion management and psychological well-being
Source: Front Psychol. 2023 Mar 3;14:1035742. doi: 10.3389/fpsyg.2023.1035742 (PMC10020588; doi:10.3389/fpsyg.2023.1035742)
Supplement: Supplementary file 1 [file Data_Sheet_1.docx]

Supplementary Material

**Supplementary Text 1.** Example answers of Internet chats

1. Examples of chats that varied in targets in context 1

Q1 *Interactor* ***(friend)***: “Have you heard that I got first prize in the competition?”

Participant (not interested in the news): “No, I didn’t 😑” (emotion expression: 80~100%)

Q3 *Interactor* ***(higher social status)***: “Have you heard that I got first prize in the competition?”

Participant (not interested in the news): “No, I didn’t 😊” (emotion expression: 0~20%)

2. Examples of chats that varied in targets in context 2

Q5 *Interactor* ***(friend)*** who broke a promise: “Sorry, I had something to do today, so I couldn’t go there.”

Participant (angry): “really 😡” (emotion expression: 80~100%)

Q7 *Interactor* ***(higher social status)*** who broke a promise: “Sorry, I had something to do today, so I couldn’t go there.”

Participant (angry): “really 😊” (emotion expression: 0~20%)

3. Examples of chats that varied in public or private settings in a positive context

Q9 *<Private chat>* Participant (honestly congratulating friends on their triumphs):

“Well done! Congratulations 🎉” (emotion expression: 80~100%)

Q10 *<Group chat in the presence of a loser>* Participant (honestly congratulating friends on their triumphs):

“Well done! Congratulations 0” (emotion expression: 80%~100%)

4. Examples of chats that varied in public or private settings in a negative context

Q11 *<Private chat>* (Participant is unsatisfied with a friend’s remarks):

“Stop saying that 😡” (emotion expression: 80~100%)

Q12 *<Group chat in the presence of other strangers>* (Participant is unsatisfied with a friend’s remarks):

“Stop saying that 😂” (emotion expression: 20~40%)

**Supplementary Table 1.** Emoji categories

| 1 | face-smiling | 21 | hand-prop | 41 | food-fruit | 61 | award-medal | 81 | household |
| --- | --- | --- | --- | --- | --- | --- | --- | --- | --- |
| 2 | face-affection | 22 | body-parts | 42 | food-vegetable | 62 | sport | 82 | other-object |
| 3 | face-tongue | 23 | person | 43 | food-prepared | 63 | game | 83 | transport-sign |
| 4 | face-hand | 24 | person-gesture | 44 | food-asian | 64 | arts & crafts | 84 | warning |
| 5 | face-neutral-skeptical | 25 | person-role | 45 | food-marine | 65 | clothing | 85 | arrow |
| 6 | face-sleepy | 26 | person-fantasy | 46 | food-sweet | 66 | sound | 86 | religion |
| 7 | face-unwell | 27 | person-activity | 47 | drink | 67 | music | 87 | zodiac |
| 8 | face-hat | 28 | person-sport | 48 | dishware | 68 | musical-instrument | 88 | av-symbol |
| 9 | face-glasses | 29 | person-resting | 49 | place-map | 69 | phone | 89 | gender |
| 10 | face-concerned | 30 | family | 50 | place-geographic | 70 | computer | 90 | other-symbol |
| 11 | face-negative | 31 | person-symbol | 51 | place-building | 71 | light & video | 91 | keycap |
| 12 | face-costume | 32 | hairstyle | 52 | place-religious | 72 | book-paper | 92 | alphanum |
| 13 | cat-face | 33 | animal-mammal | 53 | place-other | 73 | money | 93 | geometric |
| 14 | monkey-face | 34 | animal-bird | 54 | transport-ground | 74 | mail | 94 | flag |
| 15 | emotion | 35 | animal-amphibian | 55 | transport-water | 75 | writing | 95 | country-flag |
| 16 | hand-fingers-open | 36 | animal-reptile | 56 | transport-air | 76 | office | 96 | subdivision-flag |
| 17 | hand-fingers-partial | 37 | animal-marine | 57 | hotel | 77 | lock | 97 | no emoji |
| 18 | hand-single-finger | 38 | animal-bug | 58 | time | 78 | tool | 98 | emoticons |
| 19 | hand-fingers-closed | 39 | plant-flower | 59 | sky & weather | 79 | science |  |  |
| 20 | hands | 40 | plant-other | 60 | event | 80 | medical |  |  |
